# The effects of different types of prenatal exercise on neonatal outcomes: a systematic review and network meta-analysis

**DOI:** 10.3389/fphys.2026.1863589

**Published:** 2026-07-08

**Authors:** Cuiyue Kong, Sichen Zhou, Xianyang Xin, Yang Zhang

**Affiliations:** 1 Innovation Center for Integration of Sports and Medicine, Capital University of Physical Education and Sports, Beijing, China; 2 School of Wushu and Performance, Capital University of Physical Education and Sports, Beijing, China

**Keywords:** health, neonatal outcomes, network meta-analysis, pregnant women, prenatal exercise

## Abstract

**Objective:**

This study aimed to systematically evaluate and compare the effects of different types of prenatal exercise on neonatal outcomes, and to assess their relative effects and rankings for birth weight, gestational age, 1-minute Apgar score, and 5-minute Apgar score.

**Methods:**

We searched PubMed, the Cochrane Library, Embase, Scopus, and Web of Science for studies examining the effects of prenatal exercise on pregnancy and neonatal outcomes, from database inception to January 2026. According to exercise type, the interventions were classified into aerobic exercise (AE), resistance exercise (RE), mind-body exercise (MBE, referring to exercise modalities such as yoga and Pilates that emphasize breathing regulation, postural control, and mind-body coordination), and combined exercise (CE, referring to interventions composed of two or more exercise components, such as combined aerobic and resistance exercise). A network meta-analysis (NMA) was performed to synthesize the evidence.

**Results:**

A total of 61 studies involving 11,036 pregnant women were ultimately included. For the 1-minute Apgar score, mind-body exercise was associated with a higher score compared with no intervention (MD = 0.33, 95% CI: 0.18 to 0.47). In the network comparison, mind-body exercise showed a higher 1-minute Apgar score than combined exercise (MD = 0.24, 95% CI: 0.06 to 0.42). For the 5-minute Apgar score, a statistically significant increase was observed only for mind-body exercise compared with no intervention (MD = 0.18, 95% CI: 0.04 to 0.32). Regarding birth weight, mind-body exercise was associated with a higher birth weight than combined exercise (MD = 104.41, 95% CI: 1.28 to 207.54). For gestational age, no statistically significant differences were observed between any intervention and no intervention or among different interventions. The cumulative ranking probability plots showed that mind-body exercise had the highest probability of ranking first across all four outcomes. The CINeMA assessment indicated that the confidence in the evidence for this network meta-analysis was mainly moderate to low.

**Conclusion:**

The effects of different types of prenatal exercise on neonatal outcomes were not consistent. Mind-body exercise showed a relative advantage in improving Apgar scores and may have a potential effect on birth weight; however, there is insufficient evidence to indicate that it can significantly improve gestational age.

**Systematic review registration:**

https://www.crd.york.ac.uk/prospero/CRD420251241770, identifier CRD420251241770.

## 1 Introduction

Physical activity during pregnancy is one of the important modifiable factors affecting maternal and infant health, with profound implications for fetal growth and development, neonatal birth status, and long-term health outcomes (Luoto et al., 2013). Traditionally, pregnant women were advised to reduce physical activity, partly because of concerns that exercise might have adverse effects on the fetus (Schramm et al., 1996; Melzer et al., 2010; Kehler and Heinrich, 2015). However, with the accumulation of evidence-based findings, this view has changed substantially. The World Health Organization (WHO) recommends that pregnant women without contraindications engage in regular physical activity throughout pregnancy and the postpartum period, including at least 150 min of moderate-intensity aerobic physical activity per week (Bull et al., 2020). Similarly, the 2019 Canadian guideline for physical activity throughout pregnancy emphasizes that, in the absence of contraindications, prenatal physical activity should be considered a front-line strategy for reducing the risk of pregnancy complications and enhancing maternal physical and mental health, with no identified increase in adverse pregnancy or neonatal outcomes (Mottola et al., 2018). The American College of Obstetricians and Gynecologists (ACOG) also states that physical activity during pregnancy is generally associated with low risk and has been shown to benefit most pregnant women (Syed et al., 2021). Previous systematic evidence also suggested that exercise during pregnancy is beneficial for maternal health, is not associated with increased risks for the newborn, and may contribute to long-term lifestyle benefits (Nascimento et al., 2012). Existing studies further suggest that appropriate prenatal exercise may exert positive effects on fetal development by improving maternal metabolic status, promoting placental function, and optimizing the intrauterine environment (Mudd and Evenson, 2015; Chae et al., 2022; Kubler et al., 2022).

Neonatal outcomes such as birth weight, gestational age, and Apgar scores have become important endpoints for evaluating the potential effects of prenatal exercise. These indicators can not only be used to assess intrauterine fetal growth and development and early neonatal adaptation after birth, but are also closely associated with short- and long-term neonatal health (Salas et al., 2016; Marra and Radice, 2026). Specifically, birth weight reflects intrauterine fetal growth and nutritional status, while abnormal birth weight may indicate potentially adverse growth patterns and is associated with long-term health risks (Hack et al., 1995; Gaskins et al., 2010; Scifres, 2021; Shen et al., 2021). Gestational age is closely related to fetal maturity and perinatal risk stratification; the lower the gestational age, the higher the risk of related health complications (Källén et al., 2015). Apgar scores are used to evaluate the immediate postnatal condition and response to resuscitation of newborns, with lower scores being associated with an increased risk of adverse perinatal outcomes (Ehrenstein, 2009; Hodgins, 2015; Chen et al., 2022).

Although relevant guidelines clearly recommend regular physical activity during pregnancy, the actual level of physical activity among pregnant women remains generally insufficient. A study based on objective measurements showed that approximately 32% of pregnant women met the recommended physical activity level in early pregnancy, whereas this proportion decreased to approximately 12% in late pregnancy (Ruifrok et al., 2014). In addition to physiological factors such as pregnancy-related fatigue and discomfort, concerns about exercise safety, lack of time, and insufficient professional guidance are also considered common barriers limiting participation in prenatal exercise. These barriers are reflected in inadequate understanding of appropriate exercise types, intensity, and safety ranges (Evenson et al., 2009; Harrison et al., 2018). In this context, clarifying the effects of different types of prenatal exercise on neonatal outcomes may not only help optimize exercise guidance during pregnancy, but also improve the feasibility of interventions and provide a basis for developing individualized exercise prescriptions.

At present, numerous systematic reviews and meta-analyses have examined the association between prenatal exercise and maternal and neonatal outcomes. However, previous evidence has mainly focused on overall effects or on a single type of exercise, such as aerobic exercise (Davenport et al., 2018; Veisy et al., 2021; Wang et al., 2022; Andargie et al., 2025). In contrast, systematic and direct comparative evidence remains limited regarding the relative effects and priority ranking of different exercise types on key neonatal outcomes, including birth weight, gestational age, and Apgar scores. Therefore, this study aims to integrate existing evidence on the effects of different types of prenatal exercise on neonatal outcomes through a systematic review and network meta-analysis. We compared the relative effects of aerobic exercise, resistance exercise, mind-body exercise, and combined exercise, and comprehensively ranked these interventions, with the aim of providing evidence-based support for the development of individualized exercise programs during pregnancy and the optimization of perinatal health management strategies.

## 2 Study design and methods

This study was reported in accordance with the Preferred Reporting Items for Systematic Reviews and Meta-Analyses (PRISMA) guidelines (Page et al., 2021) and was registered in PROSPERO (registration number: CRD420251241770).

### 2.1 Literature search strategy

A systematic search was conducted in the PubMed, Web of Science, Scopus, Cochrane Library, and Embase electronic databases from inception to January 1, 2026. A combination of subject headings and free-text terms was used to retrieve relevant studies, and the language was restricted to English. As an example of the English search terms, the PubMed search strategy was as follows: (((((“Exercise”[Mesh])) OR (((((Physical Activity[Title/Abstract]) OR (Aerobic Exercise[Title/Abstract])) OR (Resistance Exercise[Title/Abstract])) OR (Combined Exercise[Title/Abstract]) OR (Yoga[Title/Abstract])) OR (Pilates[Title/Abstract])))) OR (((((Physical Activity[Title/Abstract]) OR (Aerobic Exercise[Title/Abstract])) OR (Resistance Exercise[Title/Abstract])) OR (Combined Exercise[Title/Abstract]) OR (Yoga[Title/Abstract])) OR (Pilates[Title/Abstract]) OR (“Exercise”[Mesh]))) AND (“Pregnancy Outcome”[Mesh] OR “Pregnancy Complications”[Mesh])) AND (randomized controlled trial[Publication Type] OR randomized[Title/Abstract] OR Clinical Trial[Title/Abstract]). The complete search strategies for each database are available in **Supplementary File 2**. In addition, to supplement potentially relevant studies that may have been missed in the database search, citation tracking was performed by screening the reference lists of the included studies and relevant reviews.

### 2.2 Inclusion criteria

Based on the PICOS framework (participants, interventions, comparators, outcomes, and study design), the inclusion criteria were as follows:

1. The study population consisted of pregnant women with singleton pregnancies, a gestational age of ≥8 weeks, and an age of ≥18 years.>
2. The intervention was regular exercise, including combined exercise (CE), mind-body exercise (MBE), aerobic exercise (AE), or resistance exercise (RE). Pregnant women were required to receive an organized exercise training program lasting at least 6 weeks. For studies with multiple exercise intervention groups, they were included as multi-arm studies, and repeated counting of shared control groups was avoided in the statistical analysis.
3. The control group was defined as no exercise intervention or usual care (N).
4. The primary outcomes included at least one of the following: 1-minute Apgar score, 5-minute Apgar score, birth weight, or gestational age.
5. The study design was a randomized controlled trial or an observational cohort study.

### 2.3 Exclusion criteria

1. Studies for which the full text or original data could not be obtained.
2. Duplicate publications or studies with unclear data descriptions.
3. Studies with unclear intervention protocols, or studies in which exercise interventions were combined with non-exercise interventions, such as diet, medication, or health education, and the independent effect of exercise could not be extracted.
4. Studies in which the control group received an exercise intervention, or studies in which the control group did not meet the definition of no exercise intervention or usual care.
5. Studies that did not report means and standard deviations, and for which means and standard deviations could not be obtained through data conversion.

### 2.4 Literature screening and data extraction

In the initial screening stage, all retrieved records were imported into EndNote X9 reference management software, and duplicate records were removed. Subsequently, two researchers (CY-K and SC-Z) independently screened the titles and abstracts to identify potentially eligible studies. Studies that met the preliminary screening criteria were then subjected to full-text assessment, and the reasons for exclusion were recorded. Any disagreements between the two researchers during the screening or full-text assessment process were resolved through discussion; if consensus could not be reached, a third researcher (YZ) made the final decision. When necessary, the corresponding authors were contacted to obtain missing or unclear data.

Data extraction was independently performed by two researchers (CY-K and XY-X) using a predesigned data extraction form. The extracted information mainly included: (1) basic study information; (2) characteristics of the study participants; (3) intervention; and control measures; and (4) outcome indicators and related data.

### 2.5 Evaluation of literature quality

Two researchers (CY-K and SC-Z) independently assessed the methodological quality of the included studies. For randomized controlled trials, the Cochrane risk-of-bias tool ROB 1 was used (Higgins et al., 2011), covering seven domains: random sequence generation, allocation concealment, blinding of participants and personnel, blinding of outcome assessment, incomplete outcome data, selective reporting, and other sources of bias. Risk-of-bias plots were generated using RevMan 5.4.1 software. For cohort studies, the Newcastle–Ottawa Scale (NOS) was used to assess study quality. This scale evaluates study quality in terms of participant selection, comparability between groups, and outcome assessment, with a maximum score of 9 points. All quality assessments were independently conducted by two researchers. Any disagreements were resolved through discussion or by consulting a third researcher (YZ).

### 2.6 Statistical methods

The network meta-analysis in this study was reported in accordance with the PRISMA-NMA statement (Hutton et al., 2015). The analysis was conducted within a frequentist framework (Salanti, 2012) using Stata 18.0 software. Studies with multiple experimental groups were included as multi-arm studies, and shared control groups were not repeatedly counted as independent evidence. First, network evidence plots were generated to assess the connectivity of the available evidence. Subsequently, the consistency of each closed loop was assessed by calculating the inconsistency factor and its 95% confidence interval (Chaimani et al., 2013). When closed loops were formed among interventions, an inconsistency model was used to test overall inconsistency. If p > 0.05, a consistency model was used for data analysis (Shim et al., 2017). In addition, the node-splitting method was applied to examine local inconsistency and to evaluate the agreement between direct and indirect comparisons. The outcomes of this study included 1-minute Apgar score, 5-minute Apgar score, birth weight, and gestational age. The surface under the cumulative ranking curve (SUCRA) (Salanti et al., 2011) was used to rank the effects of different interventions. A higher SUCRA value indicated a greater probability that the intervention would be the best intervention option (Mbuagbaw et al., 2017). In addition, funnel plots were generated to assess publication bias and small-study effects.

The certainty of evidence for the network meta-analysis results was assessed using the CINeMA framework (Nikolakopoulou et al., 2020). Evidence certainty was comprehensively evaluated across six domains: within-study bias, reporting bias, indirectness, imprecision, heterogeneity, and incoherence. The limitations in each of these six domains were judged as “none,” “some concerns,” or “major concerns,” corresponding to downgrading by 0, 1, or 2 levels, respectively. The final certainty of evidence was classified into four levels: high, moderate, low, and very low. The entire assessment process was independently completed by two researchers. Any disagreements were resolved by consulting a third researcher, after which the final judgment was determined.

## 3 Results

### 3.1 Literature search results

**Figure 1:**
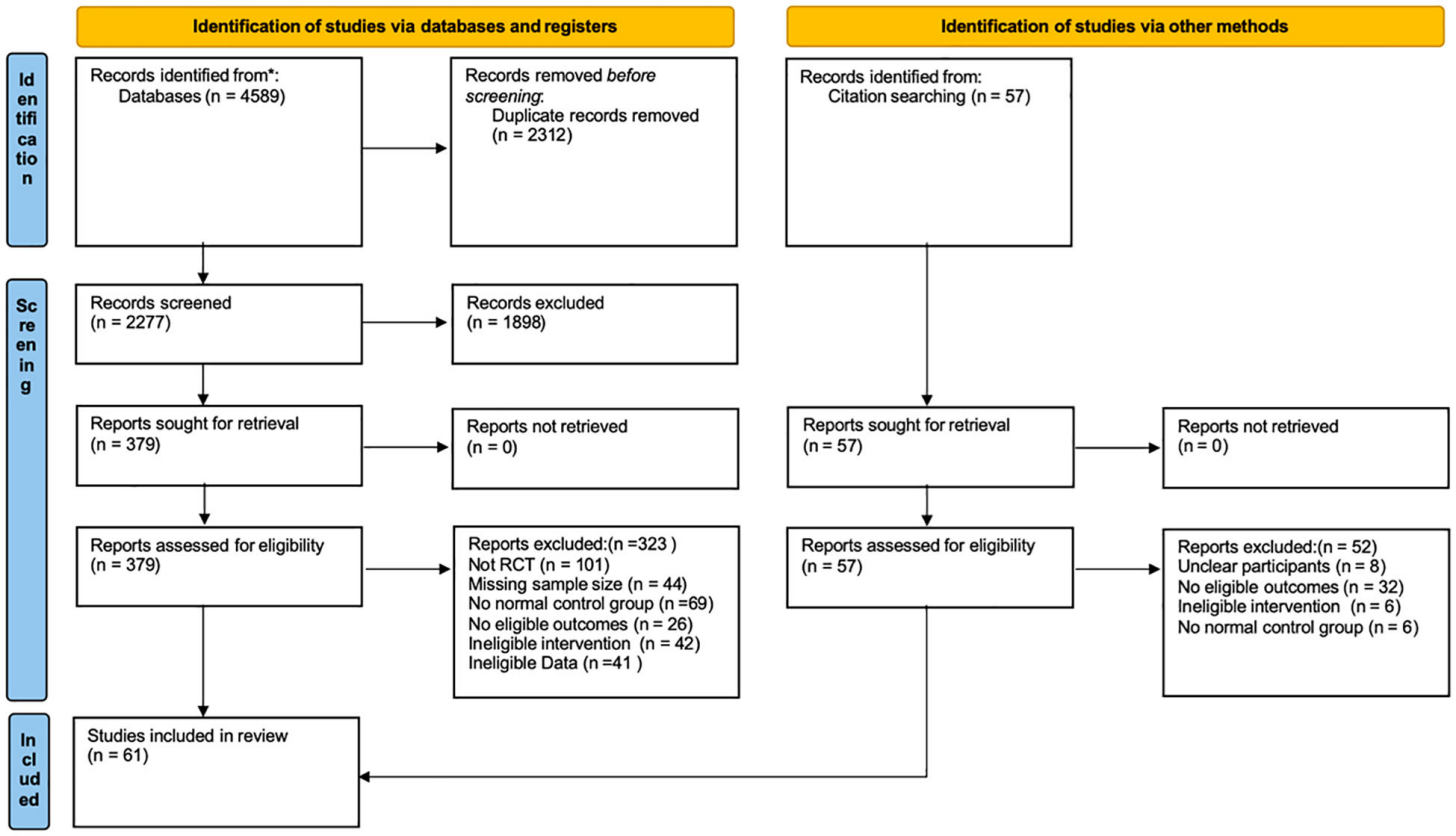
The flowchart based on PRISMA standards.

A total of 4,589 records were identified through database searches, including PubMed (n = 702), Scopus (n = 1,403), Embase (n = 1,096), Cochrane Library (n = 580), and Web of Science (n = 808). In addition, 57 records were identified through citation tracking, among which one prospective cohort study ultimately included in the review was identified through this approach. After removal of 2,312 duplicate records from the database search results, 2,277 records remained for title and abstract screening. During the initial screening stage, 1,898 records were excluded, and 379 articles proceeded to full-text retrieval and assessment, with no full texts unavailable. During the full-text assessment stage, 323 articles identified from database searches were excluded for the following reasons: ineligible study design (n = 101), missing sample size information (n = 44), absence of a normal control group (n = 69), ineligible outcomes (n = 26), ineligible interventions (n = 42), and unavailable data (n = 41). All 57 articles identified through citation tracking proceeded to full-text retrieval and assessment, with no full texts unavailable. After full-text assessment, 52 articles were excluded for the following reasons: unclear participant information (n = 8), ineligible outcomes (n = 32), ineligible interventions (n = 6), and absence of a normal control group (n = 6). Ultimately, 56 studies identified through database searches and 5 studies identified through citation tracking were included, resulting in a total of 61 studies included in the systematic review and meta-analysis (**Figure 1**).

### 3.2 Basic characteristics of the included studies

This meta-analysis included 61 studies published between 1997 and 2024 (**Supplementary File 3**), of which 60 were randomized controlled trials and one was a prospective cohort study, involving a total of 11,036 pregnant women. These studies were conducted across 20 countries/regions, including 13 in Spain, eight in Iran, seven in the United States, four each in China and Brazil, three each in Canada, Denmark, Norway, and Turkey, two each in India and New Zealand, and one each in Argentina, Australia, Japan, Kosovo, the Netherlands, Slovenia, Sweden, Thailand, and Croatia/Serbia. The exercise interventions were classified as combined exercise (CE), mind-body exercise (MBE), aerobic exercise (AE), and resistance exercise (RE). Among the included studies, 58 adopted a single exercise intervention arm, whereas three included multiple exercise intervention arms, namely RE/AE and RE/AE/CE.

### 3.3 Quality assessment of the included studies

The risk-of-bias assessment results for the included RCTs are presented in **Supplementary File 4**, and the quality assessment results for the prospective cohort study are presented in **Supplementary File 5**. Overall, most included RCTs were judged as having a low risk of bias in random sequence generation, blinding of outcome assessment, selective reporting, and other sources of bias, suggesting that the overall methodological quality of the included studies was acceptable. However, some studies were rated as having an unclear risk of bias in allocation concealment, completeness of outcome data, and selective reporting, which may reflect insufficient reporting of relevant methodological details. In addition, the proportion of high risk in blinding of participants and personnel was relatively high, which is related to the difficulty of fully blinding participants and intervention providers in exercise intervention studies. Therefore, performance bias may remain an important factor affecting the interpretation of the evidence in this study. The included prospective cohort study was assessed using the Newcastle–Ottawa Scale, with a total score of 6 points, indicating moderate study quality; however, the non-randomized study design and potential confounding factors should still be considered when interpreting the results.

### 3.4 Results of the network meta-analysis

#### 3.4.1 Network relationships

**Figure 2:**
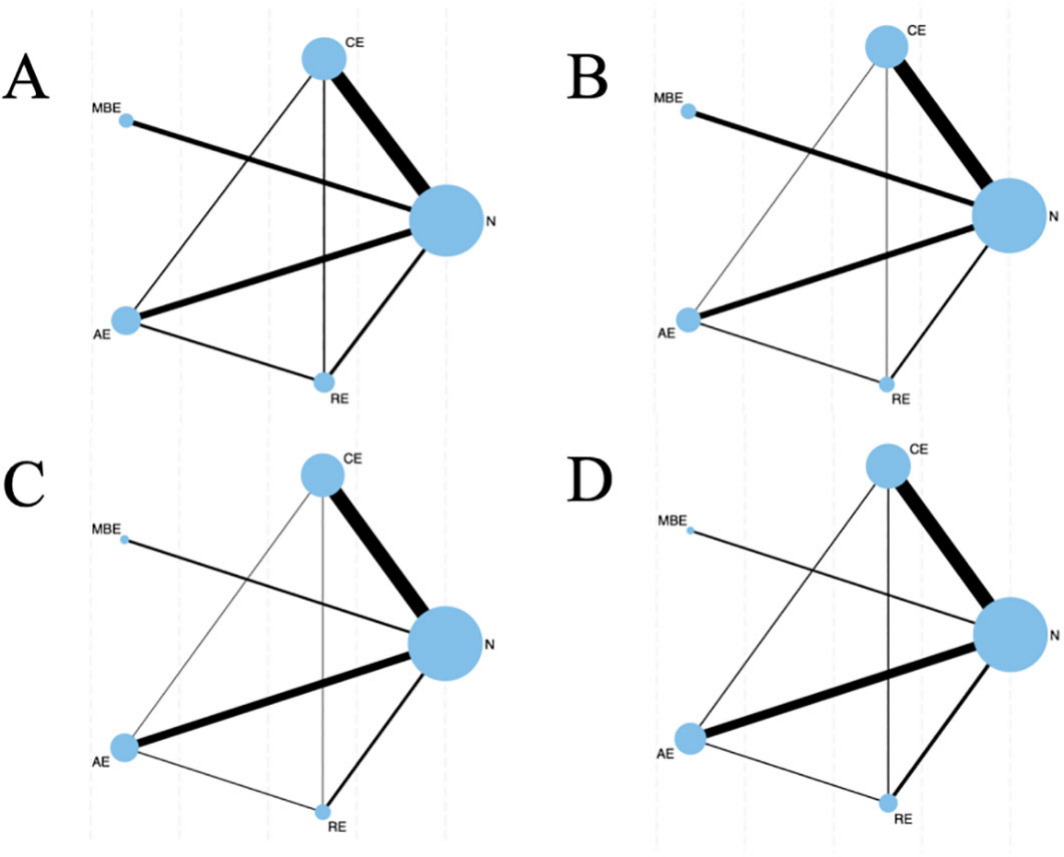
Geometry of the network. The size of the node represents the number of participants in each intervention. The thickness of the edges represents the number of studies in each treatment comparison. **(A)** 1-minute Apgar scores; **(B)** 5-minute Apgar scores; **(C)** birth weight; **(D)** gestational age. CE, combined exercise; MBE, mind-body exercise; AE, aerobic exercise; RE, resistance exercise; N, no intervention.

A total of 61 studies involving 11,036 pregnant women were included in this network meta-analysis. For the 1-minute Apgar score, 28 studies were included, comprising 13 studies on CE, 6 on MBE, 5 on AE, and 4 on RE (4,542 participants in total: 2,329 in the exercise groups and 2,213 in the control groups). For the 5-minute Apgar score, 28 studies were included, comprising 14 studies on CE, 6 on MBE, 5 on AE, and 3 on RE (4,412 participants in total: 2,227 in the exercise groups and 2,185 in the control groups). For birth weight, 54 studies were included, comprising 27 studies on CE, 6 on MBE, 14 on AE, and 7 on RE (9,970 participants in total: 5,097 in the exercise groups and 4,873 in the control groups). For gestational age, 46 studies were included, comprising 23 studies on CE, 6 on MBE, 12 on AE, and 5 on RE (8,691 participants in total: 4,381 in the exercise groups and 4,310 in the control groups). In **Figure 2**, the nodes represent different intervention types, and the size of each node represents the sample size. The lines between nodes indicate direct comparisons between two interventions, and the thickness of the lines represents the number of studies contributing to each comparison. If there is no line between two interventions, this indicates a lack of direct comparative evidence between them, and indirect comparisons need to be performed through network meta-analysis.

#### 3.4.2 Consistency check

We assessed heterogeneity and consistency in the network meta-analysis including all interventions. The global consistency test showed no significant inconsistency between direct and indirect evidence for the 1-minute Apgar score outcome (P = 0.6982); therefore, a consistency model was used for the analysis. For the 5-minute Apgar score outcome, the global consistency test also showed no significant inconsistency between direct and indirect evidence (P = 0.9980), and a consistency model was applied. For birth weight, no significant inconsistency was found between direct and indirect evidence (P = 0.1460). For gestational age, the global consistency test showed no significant inconsistency between direct and indirect evidence (P = 0.3639), and a consistency model was used for the analysis. The node-splitting analysis indicated consistency between direct and indirect evidence for all outcomes (P > 0.05), suggesting that the results were relatively robust.

#### 3.4.3 Outcome analysis

##### 3.4.3.1 1-minute Apgar score

**Figure 3:**
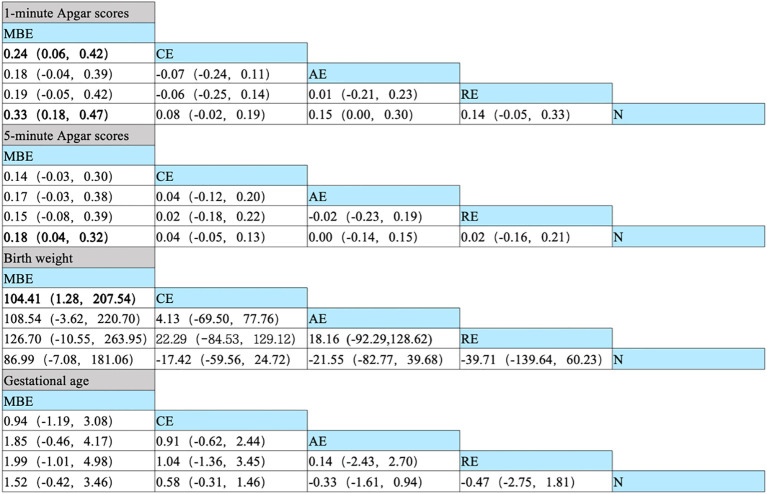
League table of network meta-analysis results for 1-minute Apgar scores, 5-minute Apgar scores, birth weight, and gestational age. Data are presented as mean differences with 95% confidence intervals.

**Figure 4:**
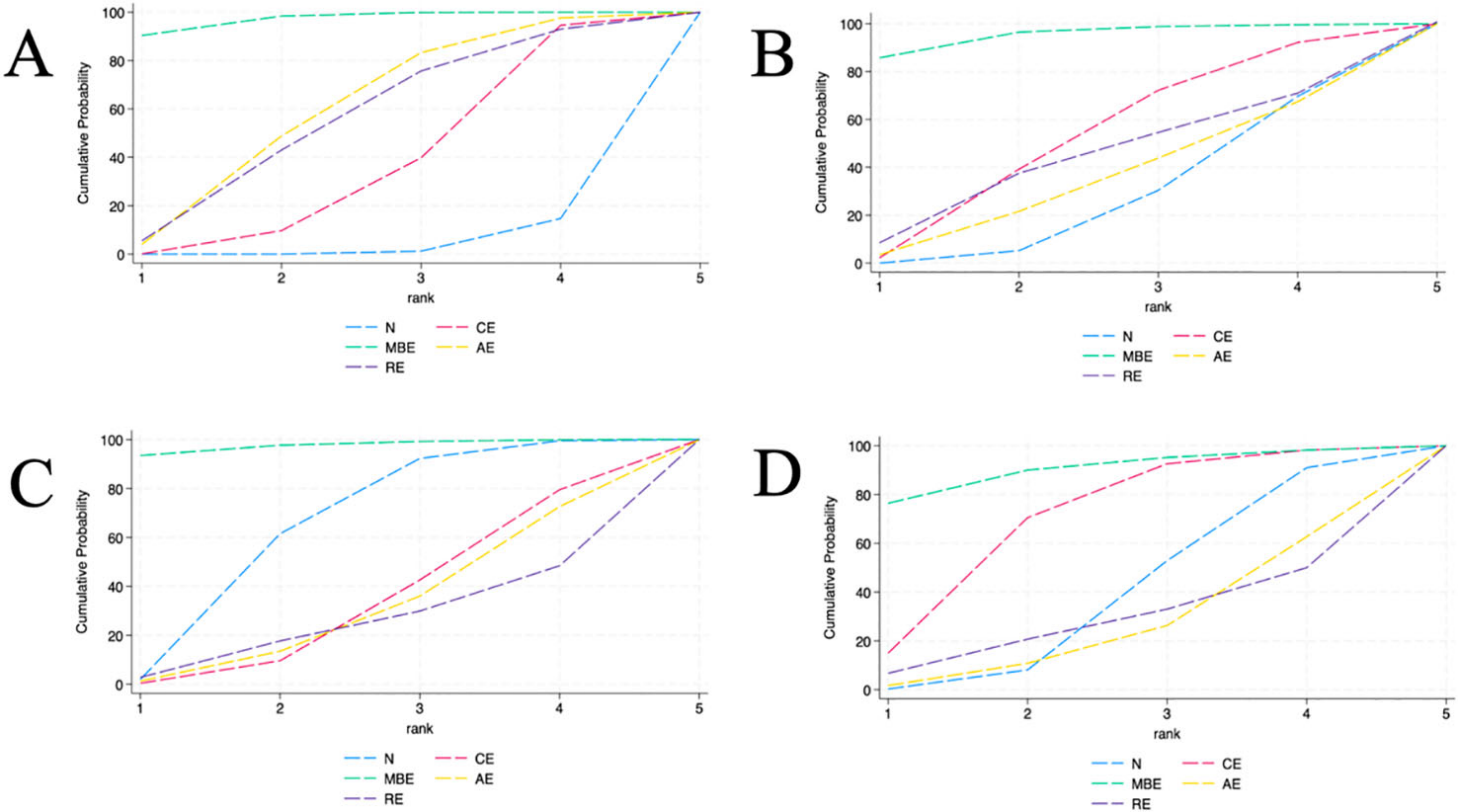
Cumulative ranking probability curves for each intervention. **(A)** 1-minute Apgar score; **(B)** 5-minute Apgar score; **(C)** birth weight; **(D)** gestational age.

The league table of the network meta-analysis showed statistically significant differences between some interventions. Compared with no intervention (N), mind-body exercise (MBE) significantly improved the neonatal 1-minute Apgar score (MD = 0.33, 95% CI: 0.18 to 0.47). In addition, MBE showed a significant advantage over combined exercise (CE) (MD = 0.24, 95% CI: 0.06 to 0.42). No statistically significant differences were observed in the remaining pairwise comparisons. The SUCRA-based probability ranking was as follows: mind-body exercise (97.1) > aerobic exercise (57.8) > resistance exercise (54.3) > combined exercise (37.1) > no intervention (3.7) (**Figures 3**, **4**; **Table 1**).

**Table 1:** Ranking of interventions according to SUCRA values.

1-minute Apgar scores		5-minute Apgar scores		Birth weight		Gestational age	
Treatment	SUCRA	Treatment	SUCRA	Treatment	SUCRA	Treatment	SUCRA
MBE	97.1	MBE	95.1	MBE	96.7	MBE	89.7
AE	57.8	CE	53.0	N	59.0	CE	69.1
RE	54.3	RE	42.2	CE	36.2	N	37.7
CE	37.1	AE	32.7	AE	34.0	RE	28.3
N	3.7	N	27.0	RE	24.1	AE	25.2

##### 3.4.3.2 5-minute Apgar score

The league table of the network meta-analysis showed that differences among different types of prenatal exercise were generally limited. Only the comparison between MBE and N reached statistical significance, suggesting that MBE may improve the neonatal 5-minute Apgar score (MD = 0.18, 95% CI: 0.04 to 0.32). No statistically significant differences were observed in the remaining pairwise comparisons. The SUCRA-based probability ranking was as follows: mind-body exercise (95.1) > combined exercise (53.0) > resistance exercise (42.2) > aerobic exercise (32.7) > no intervention (27.0) (**Figures 3**, **4**; **Table 1**).

##### 3.4.3.3 Birth weight

The league table of the network meta-analysis showed that MBE significantly increased neonatal birth weight compared with CE (MD = 104.41, 95% CI: 1.28 to 207.54). No statistically significant differences were observed in the remaining pairwise comparisons. The SUCRA-based probability ranking was as follows: mind-body exercise (96.7) > no intervention (59.0) > combined exercise (36.2) > aerobic exercise (34.0) > resistance exercise (24.1) (**Figures 3**, **4**; **Table 1**).

##### 3.4.3.4 Gestational age

The league table of the network meta-analysis showed that the overall differences in gestational age among different types of prenatal exercise were limited. None of the pairwise comparisons between interventions reached statistical significance, as the 95% CIs for all comparisons crossed 0. The SUCRA-based probability ranking was as follows: mind-body exercise (89.7) > combined exercise (69.1) > no intervention (37.7) > resistance exercise (28.3) > aerobic exercise (25.2) (**Figures 3**, **4**; **Table 1**).

#### 3.4.4 Publication bias

Publication bias was assessed for the included studies (**Supplementary File 6**). The funnel plots showed that the study points were generally symmetrically distributed, indicating a low likelihood of publication bias in the network meta-analysis.

#### 3.4.5 Evaluation of evidence quality

According to the CINeMA assessment, for improvements in neonatal 1-minute Apgar score and 5-minute Apgar score, the comparisons of CE vs N, N vs AE, N vs RE, CE vs AE, CE vs RE, and AE vs RE were mainly affected by within-study bias, leading to downgrading of the confidence in the evidence; these comparisons were therefore rated as moderate-certainty evidence. The comparison of N vs MBE was affected by both within-study bias and incoherence and was rated as low-certainty evidence. For birth weight, except for N vs AE, the remaining comparisons were mainly affected by indirectness, resulting in downgrading of the confidence in the evidence, and were rated as moderate-certainty evidence. The comparison of N vs AE was affected by both indirectness and incoherence and was rated as low-certainty evidence. For gestational age, the comparisons of CE vs N, N vs RE, and AE vs RE were mainly affected by imprecision and were rated as moderate-certainty evidence. The comparisons of CE vs AE, N vs AE, RE vs AE, and CE vs RE were affected by both imprecision and heterogeneity, leading to further downgrading of the confidence in the evidence, and were rated as low-certainty evidence. Overall, the certainty of evidence for the four neonatal outcomes ranged from moderate to low (**Supplementary Files 7**, **8**).

## 4 Discussion

This study included 61 studies involving 11,036 pregnant women and used network meta-analysis to compare the effects of different types of prenatal exercise on neonatal outcomes. Overall, the effects of different exercise types were not entirely consistent across outcomes, suggesting that the effects of prenatal exercise on neonatal outcomes may be outcome-specific. MBE showed relatively high ranking probabilities for both 1-minute Apgar score and 5-minute Apgar score, and statistically significant differences were observed in some pairwise comparisons. Specifically, for 1-minute Apgar score, MBE was associated with significantly higher scores than both N and CE. For 5-minute Apgar score, only the difference between MBE and N reached statistical significance. In terms of birth weight, MBE showed a statistically significant difference compared with CE, whereas the differences between MBE and N, AE, or RE were not statistically significant. For gestational age, no statistically significant differences were observed among the different intervention modalities. These findings suggest that the potential benefits of prenatal exercise may be more evident in early neonatal adaptation after birth, whereas its effects on gestational age and birth weight appear to be relatively limited, with no consistent superiority observed across different exercise modalities. This finding is generally consistent with previous evidence, indicating that exercise during pregnancy may help improve Apgar scores, while its effects on birth weight and gestational age are relatively limited or unstable (Sanabria-Martínez et al., 2016; Barakat et al., 2023; Silva-Jose et al., 2023; Zhang et al., 2023).

Regarding Apgar scores, this study showed that MBE had relatively high cumulative ranking probabilities for both 1-minute Apgar score and 5-minute Apgar score, although the statistical evidence for the two outcomes was not entirely consistent. For 1-minute Apgar score, the differences between MBE and both N and CE were statistically significant; for 5-minute Apgar score, only the difference between MBE and N was statistically significant. It should be noted that although some comparisons reached statistical significance, the mean differences were relatively small, with differences of approximately 0.33 and 0.18 for 1-minute Apgar score and 5-minute Apgar score, respectively. In healthy obstetric populations with generally high baseline Apgar scores, whether such small differences represent clinically meaningful improvements in early neonatal adaptation remains uncertain. Previous studies have also reported inconsistent findings regarding the effects of exercise during pregnancy on Apgar scores. A systematic review and meta-analysis by Silva-José et al., which included 50 studies, showed that physical activity during pregnancy was associated with higher 1-minute and 5-minute Apgar scores and a lower risk of NICU admission (Silva-Jose et al., 2023). However, the 2025 systematic review and meta-analysis by Andargie et al. did not observe a significant overall effect of exercise intervention on either the 1-minute or 5-minute Apgar score (Andargie et al., 2025). In addition, previous meta-analyses suggested that combined exercise produced only a slight improvement in the 1-minute Apgar score, with no significant effect on the 5-minute Apgar score (Sanabria-Martínez et al., 2016). A 2025 systematic review and meta-analysis by Li et al. on Pilates during pregnancy showed that Pilates significantly improved the 1-minute Apgar score, but had no significant effect on the 5-minute Apgar score (Li et al., 2025). Taken together, these findings suggest that the effects of exercise during pregnancy on Apgar scores may not represent a stable and consistent overall effect, but may instead be influenced by the specific exercise modality, characteristics of the study population, and baseline level of the outcome. Therefore, the relatively high ranking probabilities and partially statistically significant findings for MBE in Apgar scores, especially the difference observed in 1-minute Apgar score, should be interpreted as a favorable signal in early neonatal status scores. Whether such small improvements in scores can translate into clinically meaningful benefits in early neonatal adaptation requires further investigation.

Regarding birth weight, MBE ranked among the top interventions in terms of cumulative ranking probability. However, pairwise comparisons showed that only the difference between MBE and CE was statistically significant, whereas the differences between MBE and N, AE, or RE did not reach statistical significance. Therefore, this finding should be interpreted with caution. Previous evidence generally suggests that exercise during pregnancy has a limited overall effect on mean birth weight, and its relatively consistent benefit is mainly reflected in reducing the risk of macrosomia, without increasing the risks of low birth weight, SGA, or LGA (Wiebe et al., 2015; Davenport et al., 2018; Zhang et al., 2023). In the present study, combined exercise, aerobic exercise, and resistance exercise did not significantly affect birth weight compared with no intervention, which is broadly consistent with previous meta-analyses (Magro-Malosso et al., 2017; Pascual-Morena et al., 2021). Although MBE showed a higher birth weight than CE, this difference should not be simply interpreted as clear clinical superiority of MBE, but may instead reflect the combined effects of differences in exercise modality and study characteristics. Therefore, the relatively high ranking probability of MBE does not mean that “higher birth weight is always better”; rather, its clinical significance should be interpreted in combination with appropriate birth weight and the risk of abnormal fetal growth. Previous observational studies have also suggested a possible nonlinear relationship between physical activity during pregnancy and birth weight: moderate-intensity exercise was associated with increased neonatal birth weight, whereas high-intensity exercise was associated with reduced neonatal birth weight (Bisson et al., 2016; Bisson et al., 2017). Other studies have found no significant association between physical activity during pregnancy and neonatal birth weight (Pathirathna et al., 2019). These findings suggest that exercise type, exercise intensity, exercise volume, gestational age at the start of intervention, and baseline risk of the study population may all influence the direction and magnitude of changes in birth weight. Therefore, birth weight should not be judged simply according to whether the numerical value is higher or lower, but should be interpreted within the broader framework of appropriate fetal growth and the risk of adverse birth outcomes.

The overall effect of exercise type on pregnancy duration appeared to be limited. Previous meta-analyses have shown that exercise during pregnancy in general, as well as interventions involving aerobic exercise and combined exercise, had no significant effect on gestational age or gestational weeks and did not increase the risk of preterm birth (Di Mascio et al., 2016; Veisy et al., 2021; Wang et al., 2022; Barakat et al., 2023). The findings of the present study are consistent with this overall trend. In contrast, high-quality meta-analytic evidence specifically examining the effects of resistance exercise and mind-body exercise on gestational age remains relatively limited. Therefore, this study provides additional comparative evidence regarding the effects of different exercise types on gestational age. Some studies have suggested that physical activity during pregnancy may increase the release of catecholamines, particularly norepinephrine, thereby stimulating myometrial activity and increasing the risk of preterm birth (Di Mascio et al., 2016). However, current evidence-based findings do not support this hypothesis. An expert review by Gascoigne et al. further suggested that higher levels of physical activity are associated with a reduced risk of preterm birth, and that there is no overall evidence supporting the notion that exercise is harmful to the maintenance of pregnancy (Gascoigne et al., 2023). Therefore, the nonsignificant differences in gestational age observed in this study should not be simply interpreted as evidence that exercise is ineffective. Rather, they should be understood as indicating that prenatal exercise generally has a favorable safety profile and does not appear to exert a clear adverse effect on the normal course of pregnancy.

Notably, based on the SUCRA ranking results, MBE ranked among the top interventions across all four outcomes, particularly showing a more prominent relative advantage in the pairwise comparisons for Apgar scores. In light of previous evidence, this finding may not be attributable solely to physical activity itself, but may instead be related to the comprehensive characteristics of mind-body exercise, which combines physical activity stimulation, breathing regulation, and psychological relaxation (Govindaraj et al., 2016). Mind-body exercise represented by prenatal yoga has been shown to improve psychological states such as anxiety, depression, and perceived stress in pregnant women (Corrigan et al., 2022; Villar-Alises et al., 2023). Further physiological studies have also suggested that prenatal yoga may reduce salivary cortisol and α-amylase levels, improve subjective emotional states, and enhance parasympathetic activity and sleep quality (Kusaka et al., 2016; Hayase and Shimada, 2018). Therefore, the comprehensive regulatory effects of mind-body exercise may be more readily reflected in indicators such as Apgar scores, which capture the immediate postnatal condition of newborns. However, the advantages of MBE in birth weight and gestational age were not supported by consistent direct statistical evidence. Thus, its overall advantage across the four outcomes should be interpreted more as a potential signal based on comprehensive ranking, rather than as a stable effect that has been fully confirmed for all outcomes.

Overall, this study indicates that the effects of different types of prenatal exercise on neonatal outcomes are not consistent. Compared with birth weight and gestational age, Apgar scores, particularly the 1-minute Apgar score, may be more sensitive to changes associated with exercise interventions. Birth weight should be interpreted in relation to appropriate fetal growth and the risk of extreme birth weight, whereas gestational age is more appropriately understood from the perspective of maintaining normal pregnancy duration and confirming the safety of exercise. It should be emphasized that some exercise types were mainly studied in specific countries or regions, which may affect the transitivity assumption. In addition, the CINeMA assessment showed that the overall certainty of the relevant evidence was moderate to low, with some comparisons affected by sparse direct evidence, imprecision, and reliance on indirect evidence. Therefore, the higher ranking and favorable trends of MBE in some neonatal outcomes should not be interpreted as indicating a clear clinical superiority over other types of prenatal exercise.

Limitations of this study: First, this study included only English-language publications, which may have introduced language bias and affected the comprehensiveness of the evidence and the generalizability of the findings. Second, there was some heterogeneity in the exercise intervention protocols across the included studies, including differences in intervention duration, exercise intensity, and adherence. In addition, exercise intensity was insufficiently reported in some studies, which may have affected effect estimates and the interpretation of the results. Third, the number of studies varied across different exercise types, and direct evidence was limited for some comparisons, which may have influenced the stability of the effect estimates and SUCRA rankings. Moreover, when the network structure is relatively sparse, funnel plots have limited ability to detect publication bias. Fourth, studies on some exercise types were concentrated in specific countries, regions, or research teams. Therefore, indirect comparisons and treatment rankings may have been partly influenced by healthcare systems, cultural contexts, intervention implementation, and maternal risk characteristics, which may limit the independence and generalizability of the evidence network.

## 5 Conclusions

Overall, prenatal exercise showed a favorable safety profile and was not associated with an increased risk of adverse neonatal outcomes. However, the effects of different types of prenatal exercise on neonatal outcomes were not entirely consistent. Among them, mind-body exercise showed a certain relative advantage in improving Apgar scores, particularly the 1-minute Apgar score. Nevertheless, given that the overall certainty of the relevant evidence was moderate to low, these findings should be interpreted with caution and require further validation. Future high-quality randomized controlled trials are needed to further standardize the FITT components (frequency, intensity, time, and type) of different exercise interventions and to strengthen the reporting of outcomes such as macrosomia, low birth weight, SGA/LGA, NICU admission, and long-term follow-up outcomes, thereby improving the stability of the evidence and its clinical interpretability.

## Data Availability

The original contributions presented in the study are included in the article/**Supplementary Material**. Further inquiries can be directed to the corresponding author.
