# Supplementary material for: The effects of different types of prenatal exercise on neonatal outcomes: a systematic review and network meta-analysis
Source: Front Physiol. 2026 Jul 8;17:1863589. doi: 10.3389/fphys.2026.1863589 (PMC13388151; doi:10.3389/fphys.2026.1863589)
Supplement: Supplementary file 1 [file Supplementaryfile1.docx]

Supplementary Material

Supplementary File 1: PRISMA NMA checklist of items to include when reporting a systematic review involving a network meta-analysis

| **Section/Topic** | **Item #** | **Checklist Item** | **Reported on Page #** |
| --- | --- | --- | --- |
| **TITLE** |  |  |  |
| Title | 1 | Identify the report as a systematic review *incorporating a network meta-analysis (or related form of meta-analysis).* | 1 |
| **ABSTRACT** |  |  |  |
| Structured summary | 2 | Provide a structured summary including, as applicable:  **Background:** main objectives  **Methods:** data sources; study eligibility criteria, participants, and interventions; study appraisal; and *synthesis methods, such as network meta-analysis.*  **Results:** number of studies and participants identified; summary estimates with corresponding confidence/credible intervals; *treatment rankings may also be discussed. Authors may choose to summarize pairwise comparisons against a chosen treatment included in their analyses for brevity.*  **Discussion/Conclusions:** limitations; conclusions and implications of findings.  **Other:** primary source of funding; systematic review registration number with registry name. | 1-2 |
| **INTRODUCTION** |  |  |  |
| Rationale | 3 | Describe the rationale for the review in the context of what is already known*, including mention of why a network meta-analysis has been conducted.* | *2-3* |
| Objectives | 4 | Provide an explicit statement of questions being addressed, with reference to participants, interventions, comparisons, outcomes, and study design (PICOS). | 2-3 |
| **METHODS** |  |  |  |
| Protocol and registration | 5 | Indicate whether a review protocol exists and if and where it can be accessed (e.g., Web address); and, if available, provide registration information, including registration number. | 3 |
| Eligibility criteria | 6 | Specify study characteristics (e.g., PICOS, length of follow-up) and report characteristics (e.g., years considered, language, publication status) used as criteria for eligibility, giving rationale. *Clearly describe eligible treatments included in the treatment network, and note whether any have been clustered or* *merged into the same node (with justification).* | *4-5* |
| Information sources | 7 | Describe all information sources (e.g., databases with dates of coverage, contact with study authors to identify additional studies) in the search and date last searched. | 4 |
| Search | 8 | Present full electronic search strategy for at least one database, including any limits used, such that it could be repeated. | 4,Supplementary File 2 |
| Study selection | 9 | State the process for selecting studies (i.e., screening, eligibility, included in systematic review, and, if applicable, included in the meta-analysis). | 5 |
| Data collection process | 10 | Describe method of data extraction from reports (e.g., piloted forms, independently, in duplicate) and any processes for obtaining and confirming data from investigators. | 5 |
| Data items | 11 | List and define all variables for which data were sought (e.g., PICOS, funding sources) and any assumptions and simplifications made. | 4-5 |
| **Geometry of the network** | **S1** | Describe methods used to explore the geometry of the treatment network under study and potential biases related to it. This should include how the evidence base has been graphically summarized for presentation, and what characteristics were compiled and used to describe the evidence base to readers. | 6-7 |
| Risk of bias within individual studies | 12 | Describe methods used for assessing risk of bias of individual studies (including specification of whether this was done at the study or outcome level), and how this information is to be used in any data synthesis. | 5-6 |
| Summary measures | 13 | State the principal summary measures (e.g., risk ratio, difference in means). *Also describe the use of additional summary measures assessed, such as treatment rankings and surface under the cumulative ranking curve (SUCRA) values, as well as modified approaches used to present summary findings from meta-analyses.* | 6-7 |
| Planned methods of analysis | 14 | Describe the methods of handling data and combining results of studies for each network meta-analysis. This should include, but not be limited to:   - *Handling of multi-arm trials;* - *Selection of variance structure;* - *Selection of prior distributions in Bayesian analyses; and* - *Assessment of model fit.* | 6-7 |
| **Assessment of Inconsistency** | **S2** | Describe the statistical methods used to evaluate the agreement of direct and indirect evidence in the treatment network(s) studied. Describe efforts taken to address its presence when found. | 6-7 |
| Risk of bias across studies | 15 | Specify any assessment of risk of bias that may affect the cumulative evidence (e.g., publication bias, selective reporting within studies). | 5-6 |
| Additional analyses | 16 | Describe methods of additional analyses if done, indicating which were pre-specified. This may include, but not be limited to, the following:   - Sensitivity or subgroup analyses; - Meta-regression analyses; - *Alternative formulations of the treatment network; and* - *Use of alternative prior distributions for Bayesian analyses (if applicable).* | Not Applicable |
| **RESULTS†** |  |  |  |
| Study selection | 17 | Give numbers of studies screened, assessed for eligibility, and included in the review, with reasons for exclusions at each stage, ideally with a flow diagram. | 7-8 |
| **Presentation of network structure** | **S3** | Provide a network graph of the included studies to enable visualization of the geometry of the treatment network. | Fig. 2 |
| **Summary of network geometry** | **S4** | Provide a brief overview of characteristics of the treatment network. This may include commentary on the abundance of trials and randomized patients for the different interventions and pairwise comparisons in the network, gaps of evidence in the treatment network, and potential biases reflected by the network structure. | 9-10 |
| Study characteristics | 18 | For each study, present characteristics for which data were extracted (e.g., study size, PICOS, follow-up period) and provide the citations. | 8 |
| Risk of bias within studies | 19 | Present data on risk of bias of each study and, if available, any outcome level assessment. | 8-9,Supplementary File 4,5 |
| Results of individual studies | 20 | For all outcomes considered (benefits or harms), present, for each study: 1) simple summary data for each intervention group, and 2) effect estimates and confidence intervals. *Modified approaches may be needed to deal with information from larger networks.* | Not applicable |
| Synthesis of results | 21 | Present results of each meta-analysis done, including confidence/credible intervals. *In larger networks, authors may focus on comparisons versus a particular comparator (e.g., placebo or standard care), with full findings presented in an appendix. League tables and forest plots may be considered to summarize pairwise comparisons.* If additional summary measures were explored (such as treatment rankings), these should also be presented. | 11 |
| **Exploration for inconsistency** | **S5** | Describe results from investigations of inconsistency. This may include such information as measures of model fit to compare consistency and inconsistency models, *P* values from statistical tests, or summary of inconsistency estimates from different parts of the treatment network. | 10 |
| Risk of bias across studies | 22 | Present results of any assessment of risk of bias across studies for the evidence base being studied. | 13 |
| Results of additional analyses | 23 | Give results of additional analyses, if done (e.g., sensitivity or subgroup analyses, meta-regression analyses*, alternative network geometries studied, alternative choice of prior distributions for Bayesian analyses,* and so forth). | Not Applicable |
| **DISCUSSION** |  |  |  |
| Summary of evidence | 24 | Summarize the main findings, including the strength of evidence for each main outcome; consider their relevance to key groups (e.g., healthcare providers, users, and policy-makers). | 13-17 |
| Limitations | 25 | Discuss limitations at study and outcome level (e.g., risk of bias), and at review level (e.g., incomplete retrieval of identified research, reporting bias). *Comment on the validity of the assumptions, such as transitivity and consistency. Comment on any concerns regarding network geometry (e.g., avoidance of certain comparisons).* | 17 |
| Conclusions | 26 | Provide a general interpretation of the results in the context of other evidence, and implications for future research. | 18 |
| **FUNDING** |  |  |  |
| Funding | 27 | Describe sources of funding for the systematic review and other support (e.g., supply of data); role of funders for the systematic review. This should also include information regarding whether funding has been received from manufacturers of treatments in the network and/or whether some of the authors are content experts with professional conflicts of interest that could affect use of treatments in the network. | Not Applicable |

PICOS = population, intervention, comparators, outcomes, study design.

* Text in italics indicate wording specific to reporting of network meta-analyses that has been added to guidance from the PRISMA statement.

† Authors may wish to plan for use of appendices to present all relevant information in full detail for items in this section.

Supplementary File 2: Search strategy：

| 2.1 PubMed |
| --- |
| #1:"Exercise"[Mesh] #2:(((((((((Physical Activity[Title/Abstract]) OR (Aerobic Exercise[Title/Abstract])) OR (Resistance Exercise[Title/Abstract])) OR (Combined Exercise[Title/Abstract])) OR (Yoga[Title/Abstract])) OR (Pilates[Title/Abstract])) OR (Prenatal Exercise[Title/Abstract])) OR (Antenatal Exercise[Title/Abstract])) OR (Pregnancy Exercise[Title/Abstract])) OR (Exercise During Pregnancy[Title/Abstract]) #3:((((((((((Physical Activity[Title/Abstract]) OR (Aerobic Exercise[Title/Abstract])) OR (Resistance Exercise[Title/Abstract])) OR (Combined Exercise[Title/Abstract])) OR (Yoga[Title/Abstract])) OR (Pilates[Title/Abstract])) OR (Prenatal Exercise[Title/Abstract])) OR (Antenatal Exercise[Title/Abstract])) OR (Pregnancy Exercise[Title/Abstract])) OR (Exercise During Pregnancy[Title/Abstract]) OR ("Exercise"[Mesh]) #4:"Pregnancy Outcome"[Mesh] OR "Pregnancy Complications"[Mesh]  #5:randomized controlled trial[Publication Type] OR randomized[Title/Abstract] OR Clinical Trial[Title/Abstract]  #6:((((((((((((Physical Activity[Title/Abstract]) OR (Aerobic Exercise[Title/Abstract])) OR (Resistance Exercise[Title/Abstract])) OR (Combined Exercise[Title/Abstract])) OR (Yoga[Title/Abstract])) OR (Pilates[Title/Abstract])) OR (Prenatal Exercise[Title/Abstract])) OR (Antenatal Exercise[Title/Abstract])) OR (Pregnancy Exercise[Title/Abstract])) OR (Exercise During Pregnancy[Title/Abstract]) OR ("Exercise"[Mesh])) AND ("Pregnancy Outcome"[Mesh] OR "Pregnancy Complications"[Mesh])) AND (randomized controlled trial[Publication Type] OR randomized[Title/Abstract] OR Clinical Trial[Title/Abstract]) |

| 2.2 Embase |
| --- |
| #1'exercise'/exp OR exercise #2'physical activity' #3'aerobic exercise' #4'resistance exercise'  #5'Combined Exercise'  #6'yoga' #7'Pilates'  #8'prenatal exercise'  #9'antenatal exercise'  #10'pregnancy exercise'  #11'exercise during pregnancy'  #12'Pregnancy Outcome' #13'Pregnancy Complications'  #14'neonatal outcomes'  #15'birth outcomes' #16'randomized controlled trial' #17'rct'  #18'Clinical Trial' #19 #1 OR #2 OR #3 OR #4 OR #5 OR #6 OR #7 OR #8 OR #9 OR #10 OR #11 #20 #12 OR #13 OR #14 OR #15  #21 #16 OR #17 OR #18 #22 #19 AND #20 AND #21 |

| 2.3 Cochrane library |
| --- |
| #1:MeSH descriptor: [Exercise] explode all trees  #2:("exercise":ti,ab,kw OR "Physical Activity":ti,ab,kw OR "Aerobic Exercise":ti,ab,kw OR "Resistance Exercise":ti,ab,kw OR "Combined Exercise":ti,ab,kw OR "Yoga":ti,ab,kw OR "Pilates":ti,ab,kw)  #3:("Prenatal Exercise":ti,ab,kw OR "Antenatal Exercise":ti,ab,kw OR "Pregnancy Exercise":ti,ab,kw OR "Exercise During Pregnancy":ti,ab,kw)  #4:#1 OR #2 OR #3 #5:("Pregnancy Outcome":ti,ab,kw OR "Pregnancy Complications":ti,ab,kw OR "neonatal outcomes":ti,ab,kw OR "birth outcomes":ti,ab,kw) #6:("Randomized Controlled Trial":ti,ab,kw OR "RCT":ti,ab,kw OR "Clinical Trial":ti,ab,kw)  #7: #4 AND #5 AND #6 |

| 2.4 Scopus |
| --- |
| #1:TITLE-ABS-KEY ( "Exercise" OR "Physical Activity" OR "Aerobic Exercise" OR "Resistance Exercise" OR "Combined Exercise" OR "Yoga" OR "Pilates" OR "Prenatal Exercise" OR "Antenatal Exercise" OR "Pregnancy Exercise" OR "Exercise During Pregnancy" )  #2:TITLE-ABS-KEY ( "Pregnancy Outcome" OR "Pregnancy Complications" OR "neonatal outcomes" OR "birth outcomes" )  #3:TITLE-ABS-KEY ( "randomized controlled trial" OR "randomized" OR "Clinical Trial" OR "placebo" )  #4: #1 AND #2 AND #3 |

| 2.5 Web of science |
| --- |
| TS=("Exercise" OR "Physical Activity" OR "Aerobic Exercise" OR "Resistance Exercise" OR "Combined Exercise" OR "Yoga" OR "Pilates" OR "Prenatal Exercise" OR "Antenatal Exercise" OR "Pregnancy Exercise" OR "Exercise During Pregnancy") AND TS=("Pregnancy Complications" OR "Pregnancy Outcome" OR "neonatal outcomes" OR "birth outcomes")  AND TS=("Randomized Controlled Trial" OR "RCT" OR "randomized trial" OR "controlled trial" OR "Clinical Trial") |

Supplementary File 3:Characteristics of the included studies

|  | Country | Intervention Group | | | | | Control Group | | | Outcomes |
| --- | --- | --- | --- | --- | --- | --- | --- | --- | --- | --- |
|  |  | Sample Size | Age | Intervention type | Intervention frequent | Duration (week) | Sample Size | Age | Intervention type |  |
| Barakat 2013 | ESP | 210 | 31±3 | CE | 3 | 28 | 218 | 31±4 | N | ①②③④ |
| Cordero 2015 | ESP | 101 | 33.6±4.1 | CE | 3 | 26 | 156 | 32.9±4.5 | N | ③④ |
| Stafne 2012 | NOR | 429 | 30.5±4.4 | CE | 3 | 12 | 426 | 30.4±4.3 | N | ③④ |
| Murtezani 2014 | KOS | 30 | 26.9±4.7 | CE | 3 | 26 | 33 | 25.7±5.1 | N | ①②③④ |
| Hui 2014 | CAN | 57 | 31±3.54 | CE | 3～5 | 16～17 | 56 | 30.5±5.52 | N | ③④ |
| Hui 2012 | CAN | 102 | 30.1±5.2 | CE | 3～5 | 16 | 88 | 28.7±5.9 | N | ③④ |
| Oostdam 2012 | NED | 52 | 30.8±5.2 | CE | 2 | 16 | 53 | 30.1±4.5 | N | ③④ |
| Chauhan 2024 | IND | 100 | 24.25±4.01 | CE | 5 | 18 | 100 | 24.49±3.97 | N | ①②③ |
| Price 2012 | USA | 31 | 30.5±5 | CE | 4 | 24 | 31 | 31.25±3.36 | N | ①②③ |
| Barakat 2018 | ESP | 176 | 31.2±4.56 | CE | 3 | 27～30 | 149 | 30.3±4.4 | N | ①②③④ |
| Haakstad 2011 | NOR | 52 | 31.77±3.7 | CE | 2 | 12 | 53 | 27.6±7.5 | N | ①②③④ |
| Brik 2019 | ESP | 42 | 33.4±3.2 | CE | 3 | 29 | 43 | 32.7±4.4 | N | ③④ |
| Nascimento 2011 | BRA | 39 | 29.7±6.8 | CE | 3 | 16~26 | 41 | 30.9±5.9 | N | ③④ |
| Barakat 2016 | ESP | 382 | 31.6±4.2 | CE | 3 | 29～30 | 383 | 31.8±4.5 | N | ③④ |
| Yang 2024 | CHN | 161 | 29.25±4.09 | CE | 3 | 29～30 | 147 | 29.99±3.91 | N | ④ |
| Perales 2014 | ESP | 83 | 31.4±3.7 | CE | 3 | 30 | 83 | 31.8±4 | N | ①②③④ |
| Roland 2023 | DNK | 74 | 31.1±4.3 | CE | 3 | 28～31 | 34 | 32±4.6 | N | ③④ |
| Ruiz 2013 | ESP | 481 | 31.6±4 | CE | 3 | 29～30 | 481 | 31.9±4 | N | ①②③④ |
| Vinter 2011 | DNK | 150 | 29±3.54 | CE | 3 | 24～30 | 154 | 29±3.54 | N | ③ |
| Silva-Jose 2022 | ESP | 69 | 33.83±3.87 | CE | 3 | 29～32 | 70 | 33.41±5.91 | N | ①②③④ |
| Barakat 2012 | ESP | 138 | 31.4±3.2 | CE | 3 | 29～33 | 152 | 31.7±4.5 | N | ①②③④ |
| Blanque 2019 | ESP | 65 | 32.12±4.43 | CE | 3 | 17～18 | 64 | 30.58±4.75 | N | ③④ |
| Bacchi 2017 | ARG | 49 | 30.4±4 | CE | 3 | 26～29 | 62 | 31±5 | N | ①②③④ |
| Garshasbi 2005 | IRN | 107 | 26.27±4.87岁 | CE | 3 | 12 | 105 | 26.48±4.43 | N | ③④ |
| Barakat 2012 | ESP | 40 | 32±4 | CE | 3 | 30～32 | 43 | 31±3 | N | ①②③④ |
| Barakat 2011 | ESP | 34 | 31±3 | CE | 3 | 30～32 | 33 | 30±3 | N | ①②③④ |
| Marquez 2000 | USA | 9 | 31.3±3 | CE | 3 | 15 | 6 | 27.8±3.1 | N | ②③ |
| Johannessen 2021 | NOR | 383 | 30.6±4.3 | CE | 3 | 12 | 339 | 30.6±4.2 | N | ③ |
| Jahdi 2017 | IRN | 30 | 26.6±3.7 | MBE | 3 | 12 | 30 | 28.23±5.05 | N | ①②③ |
| Yekefallah 2021 | IRN | 35 | 27.45±4.5 | MBE | 2 | 12 | 35 | 26.82±4.38 | N | ①②③④ |
| Mohyadin 2020 | IRN | 42 | 25.07±4.05 | MBE | 2 | 9～11 | 42 | 24.07±3.86 | N | ①②④ |
| Chuntharapat 2008 | THA | 33 | 25.43±4.59 | MBE | 3 | 9～11 | 33 | 24.58±4.27 | N | ③ |
| Esencan 2023 | TUR | 30 |  | MBE | 2 | 9～11 | 60 |  | N | ④ |
| Kuder 2024 | SVN | 106 | 29.6±3.9 | MBE | 2 | 26～27 | 108 | 28.4±4.5 | N | ③ |
| Pais 2021 | IND | 61 | 27.9 ± 3.39 | MBE | 2 | 18～22 | 63 | 27.8 ± 3.52 | N | ①②③④ |
| Buran 2024 | TUR | 63 | 29.5±2.57 | MBE | 2 | 8 | 59 | 29.09±4.58 | N | ④ |
| Ghandali 2021 | IRN | 51 | 25.16±4.41 | MBE | 2 | 8 | 52 | 23.81±4.3 | N | ①② |
| Aktan 2021 | TUR | 21 | 27.52±3.88 | MBE | 2 | 8 | 22 | 25.5±4.19 | N | ①②③④ |
| Wang 2017 | CHN | 132 | 32.14±4.47 | AE | 3 | 27 | 133 | 32.5±4.91 | N | ①②③④ |
| Kong 2014 | USA | 18 | 27.4±4.05 | AE | 5 | 20 | 19 | 26.54±3.79 | N | ①②③④ |
| Taniguchi 2016 | JPN | 54 | 28.5±3.5 | AE | 3 | 10 | 53 | 29.5±3.0 | N | ③④ |
| Guelfi 2016 | AUS | 84 | 33.6±4.1 | AE | 3 | 14 | 85 | 33.8±3.9 | N | ③④ |
| Lemoyne 2016 | CAN | 10 | 28.50±3.37 | AE | 3 | 26 | 8 | 27.75±3.41 | N | ③④ |
| Renault 2014 | DNK | 125 | 30.9±4.9 | AE | 7 | 26~29 | 134 | 31.3±42 | N | ④ |
| Nobles 2015 | USA | 124 | 25.91±5.93 | AE | 3 | 12 | 127 | 26.7±5.74 | N | ③④ |
| Ghodsi 2014 | IRN | 40 | 23.4±3.69 | AE | 3 | 14~20 | 40 | 23.28±3.94 | N | ③ |
| Avery 1997 | USA | 15 | 32.2±4.9 | AE | 3~4 | 14~20 | 14 | 30.4±5.1 | N | ③④ |
| Melo 2012 | BRA | 114 | 25.4±5.56 | AE | 3~4 | 20~27 | 57 | 24±5.4 | N | ③ |
| Tomić 2013 | HRV/SRB | 166 | 28.9 | AE | 3 | 32 | 168 | 29.2±3.2 | N | ③④ |
| Shojaei 2021 | IRN | 49 | 25.12±4.37 | AE | 4 | 6 | 51 | 25.31±4.72 | N | ①② |
| Seneviratne 2016 | NZL | 38 | 18~40 | AE | 3～5 | 16 | 37 | 18~40 | N | ①②③④ |
| Toosi 2016 | IRN | 60 | 23.7±4.3 | AE | 3 | 8 | 60 | 24.2±3.4 | N | ①②③④ |
| Cavalcante 2009 | BRA | 33 |  | AE | 3 | 16 | 37 |  | N | ③ |
| Hopkins 2010 | NZL | 47 | 31±3 | AE | 5 | 20 | 37 | 29±4 | N | ③④ |
| Xie 2022 | CHN | 43 | 31.84±5.19 | RE | 3 | 6 | 46 | 31.35±4.72 | N | ①②③ |
| Xie 2022 |  | 43 | 31.47±4.06 | AE | 3 | 6 |  |  |  |  |
| Clapp 2000 | USA | 22 | 31 ± 1 | RE | 3～5 | 32 | 24 | 31 ± 1 | N | ③ |
| Barros 2010 | BRA | 32 | 31.81±4.87 | RE | 3 | 7 | 32 | 32.4±5.4 | N | ③④ |
| Barakat 2009 | ESP | 72 | 30.4±2.9 | RE | 3 | 26 | 70 | 29.5±3.7 | N | ①②③④ |
| Fieril 2015 | SWE | 38 | 30.8±3.6 | RE | 2 | 12 | 34 | 28.5±5.1 | N | ③④ |
| Jevtovic 2023 | USA | 10 | 30.5±4.6 | AE | 3~4 | 24 | 11 | 30.6±3.5 | N | ①②③④ |
| Jevtovic 2023 |  | 11 | 31.92±3.2 | RE | 3~4 | 24 |  |  |  |  |
| Jevtovic 2023 |  | 9 | 28.7±1.8 | CE | 3~4 | 24 |  |  |  |  |
| Zeng 2024 | CHN | 36 | 30.14±2.24 | AE | 3~4 | 12 | 34 | 30.6±2.08 | N | ①③④ |
| Zeng 2024 |  | 42 | 29.67±2.75 | RE | 3~4 | 12 |  |  |  |  |
| Zeng 2024 |  | 33 | 30.21±2.36 | CE | 3~4 | 12 |  |  |  |  |

Supplementary File 4:Graph of Cochrane risk bias assessment.A:Risks of bias graph.B:Risks of bias summary.


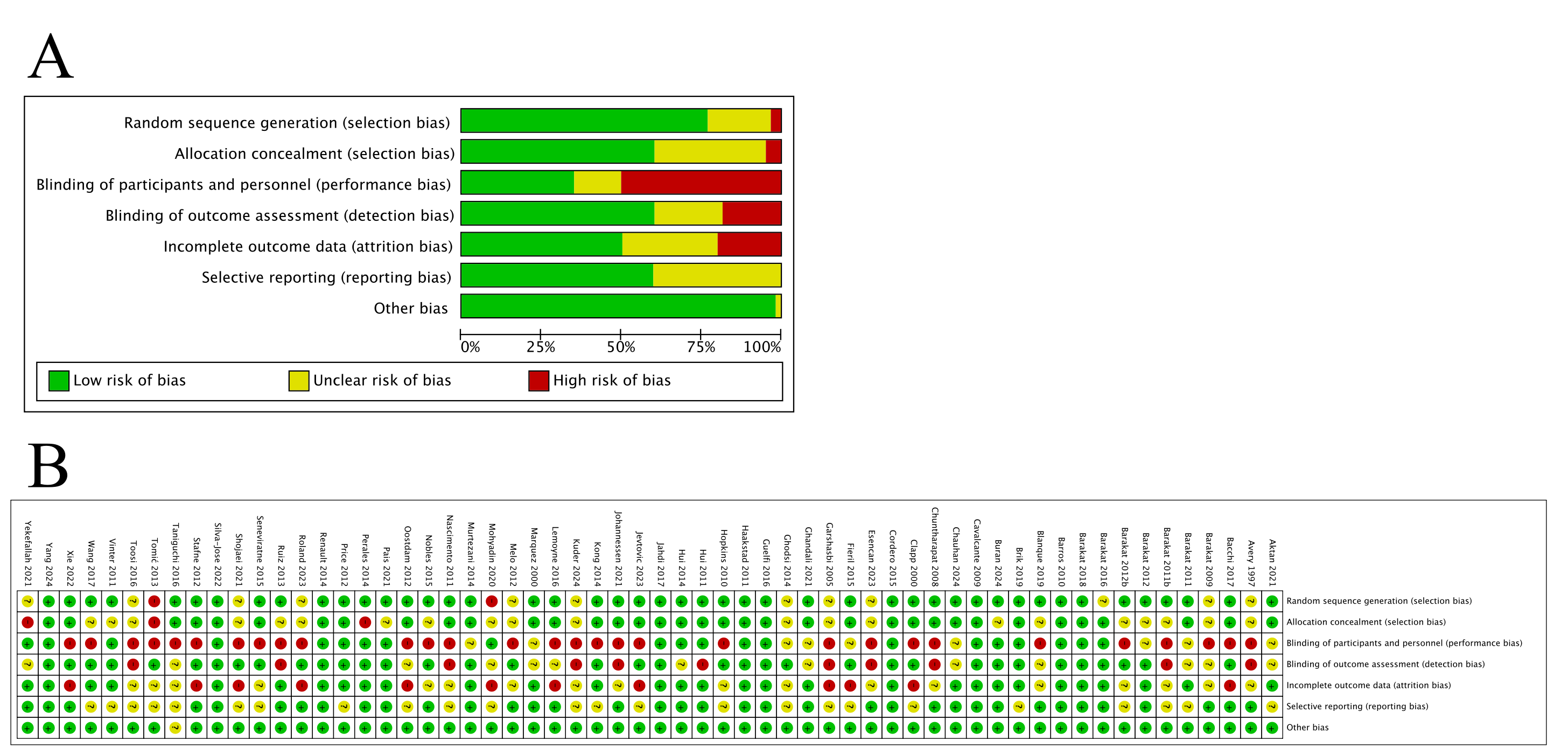


Supplementary File 5：Quality assessment of the observational studies with NOS scale.

| Study | Selection | | | Comparability | | | Outcome | | | Total  score |
| --- | --- | --- | --- | --- | --- | --- | --- | --- | --- | --- |
|  | Representative- ness | Selection of  non-exposed | Ascertainment  of exposure | Outcome not  present at start | Comparability on most important  factors | Comparability on other risk factors | Assessment  of outcome | Long enough follow-up | Adequacy  of follow-up |  |
| Zeng , et al (2024) | 1 | 1 | 1 | 1 | 0 | 0 | 1 | 1 | 0 | 6 |

Supplementary File 6: Network meta-analysis funnel plots.A: 1-minute Apgar score,B:5-minute Apgar score, C:Birth weight，D:Gestational age.

| 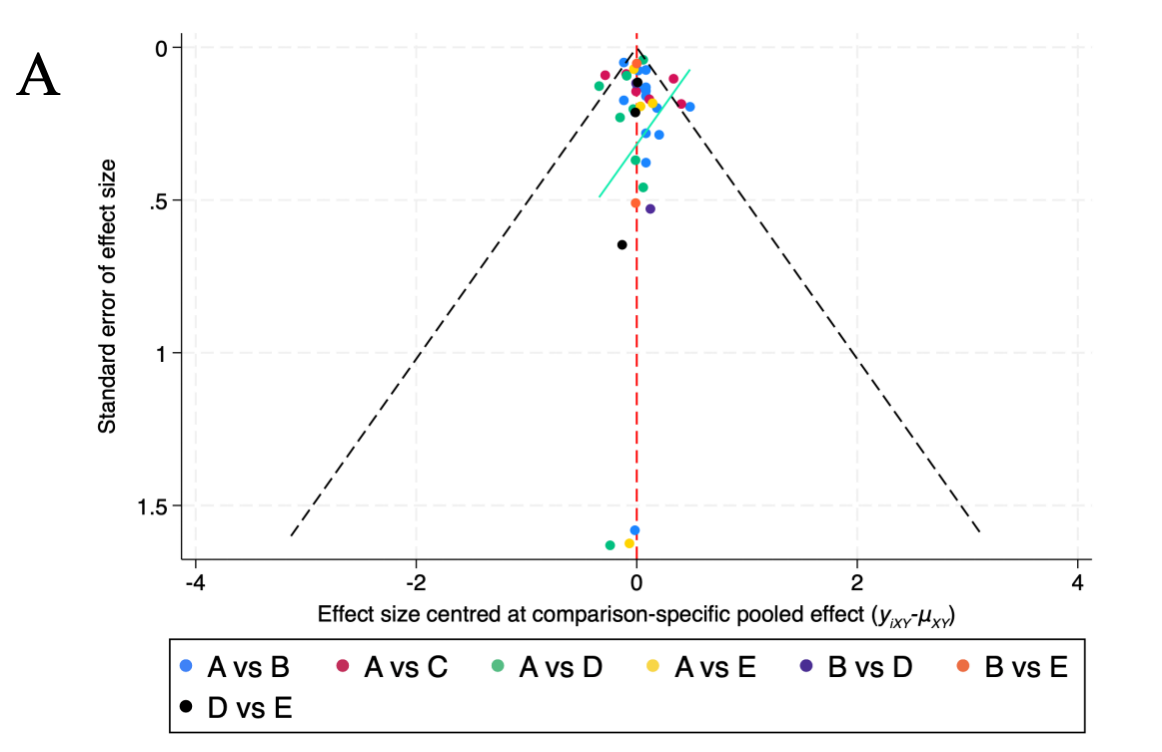 |
| --- |
| 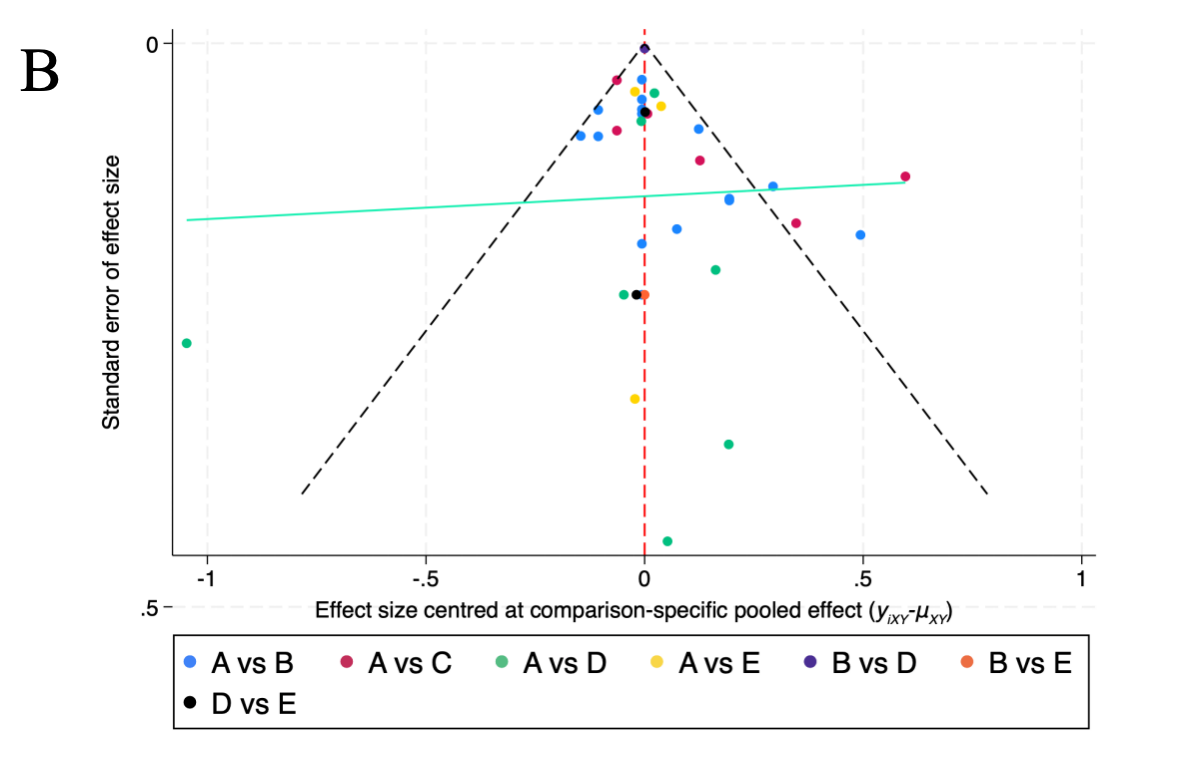 |
| 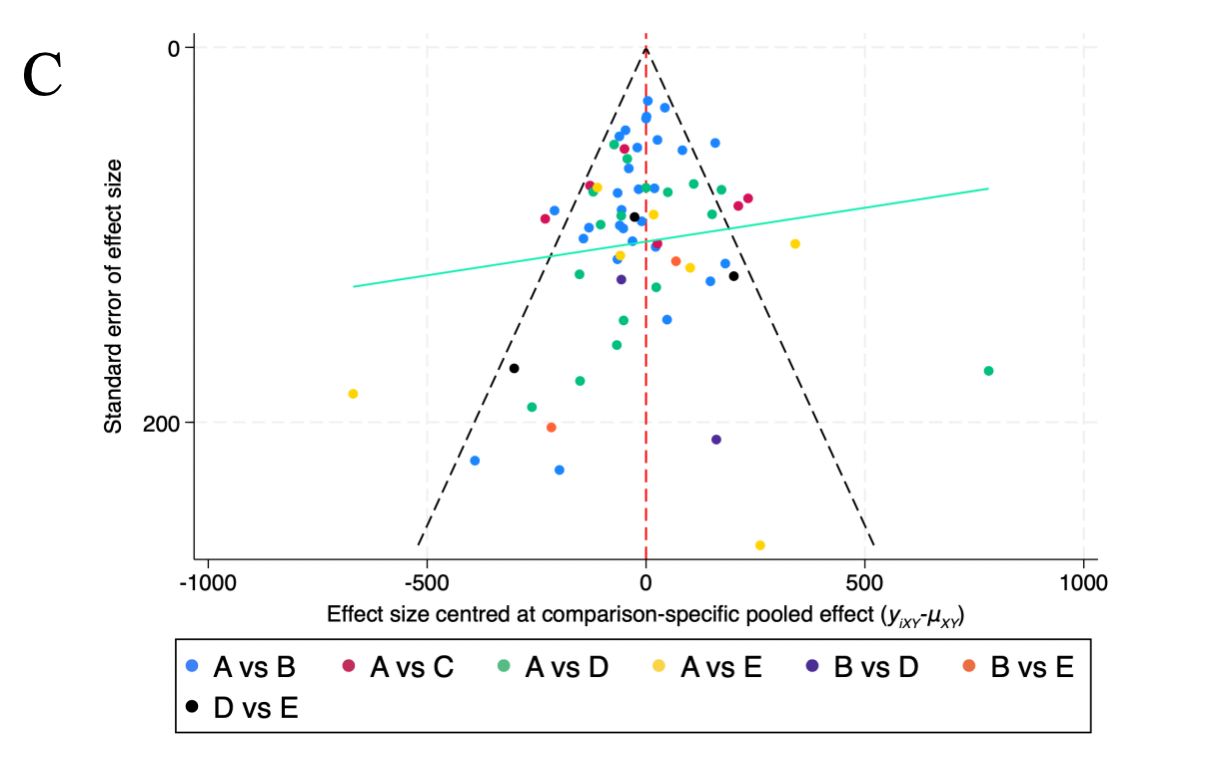 |
| 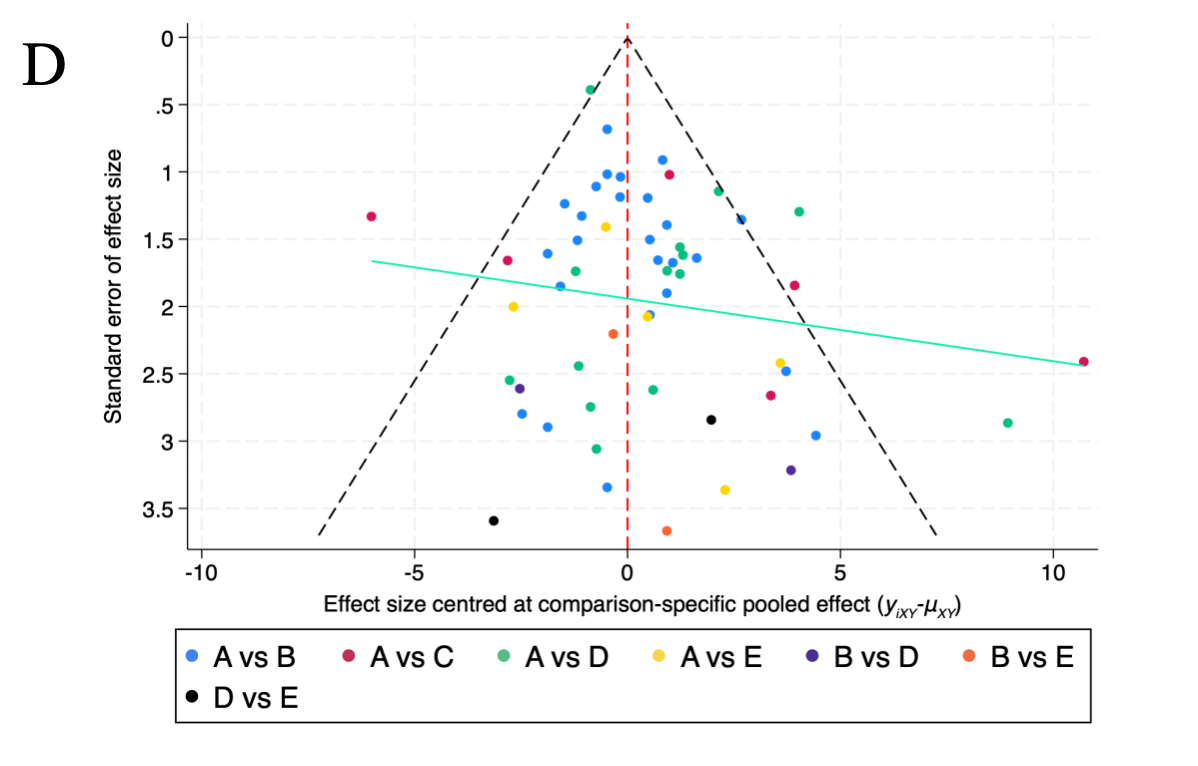 |

Supplementary File 7: CINeMA tool for evaluating the effects of different prenatal exercise modalities on neonatal outcomes.

| 7.1 CINeMA tool for evaluating the effects of different prenatal exercise modalities on neonatal 1-minute Apgar score. | | | | | | |
| --- | --- | --- | --- | --- | --- | --- |
|  | Quality assessment | | | | | |
|  | Within-study bias | Reporting bias | Indirectness | Imprecision | Heterogeneity | Incoherence |
| CE vs N | No concerns | Low risk | No concerns | No concerns | No concerns | No concerns |
| N vs MBE | No concerns | Low risk | No concerns | No concerns | No concerns | Major concerns |
| N vs AE | No concerns | Low risk | No concerns | No concerns | No concerns | No concerns |
| N vs RE | No concerns | Low risk | No concerns | No concerns | No concerns | No concerns |
| CE vs AE | No concerns | Low risk | No concerns | No concerns | No concerns | No concerns |
| CE vs RE | No concerns | Low risk | No concerns | No concerns | No concerns | No concerns |
| AE vs RE | No concerns | Low risk | No concerns | No concerns | No concerns | No concerns |

| 7.2 The CINeMA tool for evaluating the effects of different prenatal exercise modalities on neonatal 5-minute Apgar score. | | | | | | |
| --- | --- | --- | --- | --- | --- | --- |
|  | Quality assessment | | | | | |
|  | Within-study bias | Reporting bias | Indirectness | Imprecision | Heterogeneity | Incoherence |
| CE vs N | No concerns | Low risk | No concerns | No concerns | No concerns | No concerns |
| N vs MBE | No concerns | Low risk | No concerns | No concerns | No concerns | Major concerns |
| N vs AE | No concerns | Low risk | No concerns | No concerns | No concerns | No concerns |
| N vs RE | No concerns | Low risk | No concerns | No concerns | No concerns | No concerns |
| CE vs AE | No concerns | Low risk | No concerns | No concerns | No concerns | No concerns |
| CE vs RE | No concerns | Low risk | No concerns | No concerns | No concerns | No concerns |
| AE vs RE | No concerns | Low risk | No concerns | No concerns | No concerns | No concerns |

| 7.3 CINeMA tool for evaluating the effects of different prenatal exercise modalities on neonatal birth weight. | | | | | | |
| --- | --- | --- | --- | --- | --- | --- |
|  | Quality assessment | | | | | |
|  | Within-study bias | Reporting bias | Indirectness | Imprecision | Heterogeneity | Incoherence |
| CE vs N | No concerns | Low risk | No concerns | Major concerns | No concerns | No concerns |
| N vs MBE | No concerns | Low risk | No concerns | Major concerns | No concerns | Major concerns |
| N vs AE | No concerns | Low risk | No concerns | Major concerns | No concerns | No concerns |
| N vs RE | No concerns | Low risk | No concerns | Major concerns | No concerns | No concerns |
| CE vs AE | No concerns | Low risk | No concerns | Major concerns | No concerns | No concerns |
| CE vs RE | No concerns | Low risk | No concerns | Major concerns | No concerns | No concerns |
| AE vs RE | No concerns | Low risk | No concerns | Major concerns | No concerns | No concerns |

| 7.4 CINeMA tool for evaluating the effects of different prenatal exercise modalities on neonatal gestational age. | | | | | | |
| --- | --- | --- | --- | --- | --- | --- |
|  | Quality assessment | | | | | |
|  | Within-study bias | Reporting bias | Indirectness | Imprecision | Heterogeneity | Incoherence |
| CE vs N | No concerns | Low risk | No concerns | Some concerns | Some concerns | No concerns |
| N vs MBE | No concerns | Low risk | No concerns | Some concerns | Some concerns | Major concerns |
| N vs AE | No concerns | Low risk | No concerns | Some concerns | Some concerns | No concerns |
| N vs RE | No concerns | Low risk | No concerns | Major concerns | No concerns | No concerns |
| CE vs AE | No concerns | Low risk | No concerns | Some concerns | Some concerns | No concerns |
| CE vs RE | No concerns | Low risk | No concerns | Major concerns | No concerns | No concerns |
| AE vs RE | No concerns | Low risk | No concerns | Major concerns | No concerns | No concerns |

Supplementary File 8 :Quality of Evidence Evaluation for the effects of different prenatal exercise modalities on neonatal outcomes.

| 8.1 Results of a Quality of Evidence Evaluation of Different Prenatal Exercise Modalities on Neonatal 1-minute Apgar score. | | | | | | | | |
| --- | --- | --- | --- | --- | --- | --- | --- | --- |
|  | Number of studies | Within-study bias | Reporting bias | Indirectness | Imprecision | Heterogeneity | Incoherence | Quality of Evidence |
| CE vs N | 15 | -1 | 0 | 0 | 0 | 0 | 0 | Moderate |
| N vs MBE | 6 | -1 | 0 | 0 | 0 | 0 | -1 | Low |
| N vs AE | 8 | -1 | 0 | 0 | 0 | 0 | 0 | Moderate |
| N vs RE | 4 | -1 | 0 | 0 | 0 | 0 | 0 | Moderate |
| CE vs AE | 2 | -1 | 0 | 0 | 0 | 0 | 0 | Moderate |
| CE vs RE | 2 | -1 | 0 | 0 | 0 | 0 | 0 | Moderate |
| AE vs RE | 3 | -1 | 0 | 0 | 0 | 0 | 0 | Moderate |

| 8.2 Results of a Quality of Evidence Evaluation of Different Prenatal Exercise Modalities on Neonatal 5-minute Apgar score. | | | | | | | | |
| --- | --- | --- | --- | --- | --- | --- | --- | --- |
|  | Number of studies | Within-study bias | Reporting bias | Indirectness | Imprecision | Heterogeneity | Incoherence | Quality of Evidence |
| CE vs N | 15 | -1 | 0 | 0 | 0 | 0 | 0 | Moderate |
| N vs MBE | 6 | -1 | 0 | 0 | 0 | 0 | -1 | Low |
| N vs AE | 7 | -1 | 0 | 0 | 0 | 0 | 0 | Moderate |
| N vs RE | 3 | -1 | 0 | 0 | 0 | 0 | 0 | Moderate |
| CE vs AE | 1 | -1 | 0 | 0 | 0 | 0 | 0 | Moderate |
| CE vs RE | 1 | -1 | 0 | 0 | 0 | 0 | 0 | Moderate |
| AE vs RE | 2 | -1 | 0 | 0 | 0 | 0 | 0 | Moderate |

| 8.3 Results of a Quality of Evidence Evaluation of Different Prenatal Exercise Modalities on Neonatal Birth Weight. | | | | | | | | |
| --- | --- | --- | --- | --- | --- | --- | --- | --- |
|  | Number of studies | Within-study bias | Reporting bias | Indirectness | Imprecision | Heterogeneity | Incoherence | Quality of Evidence |
| CT vs AT | 29 | 0 | 0 | -1 | 0 | 0 | 0 | Moderate |
| N vs AT | 6 | 0 | 0 | -1 | 0 | 0 | -1 | Low |
| RT vs AT | 17 | 0 | 0 | -1 | 0 | 0 | 0 | Moderate |
| CT vs N | 7 | 0 | 0 | -1 | 0 | 0 | 0 | Moderate |
| CT vs RT | 2 | 0 | 0 | -1 | 0 | 0 | 0 | Moderate |
| N vs RT | 2 | 0 | 0 | -1 | 0 | 0 | 0 | Moderate |
| AE vs RE | 3 | 0 | 0 | -1 | 0 | 0 | 0 | Moderate |

| 8.4 Results of a Quality of Evidence Evaluation of Different Prenatal Exercise Modalities on Neonatal Gestational Age. | | | | | | | | |
| --- | --- | --- | --- | --- | --- | --- | --- | --- |
|  | Number of studies | Within-study bias | Reporting bias | Indirectness | Imprecision | Heterogeneity | Incoherence | Quality of Evidence |
| CT vs AT | 25 | 0 | 0 | 0 | -1 | -1 | 0 | Low |
| N vs AT | 6 | 0 | 0 | 0 | -1 | -1 | 0 | Low |
| RT vs AT | 14 | 0 | 0 | 0 | -1 | -1 | 0 | Low |
| CT vs N | 5 | 0 | 0 | 0 | -1 | 0 | 0 | Moderate |
| CT vs RT | 2 | 0 | 0 | 0 | -1 | -1 | 0 | Low |
| N vs RT | 2 | 0 | 0 | 0 | -1 | 0 | 0 | Moderate |
| AE vs RE | 2 | 0 | 0 | 0 | -1 | 0 | 0 | Moderate |

Supplementary File 9:Forest plots


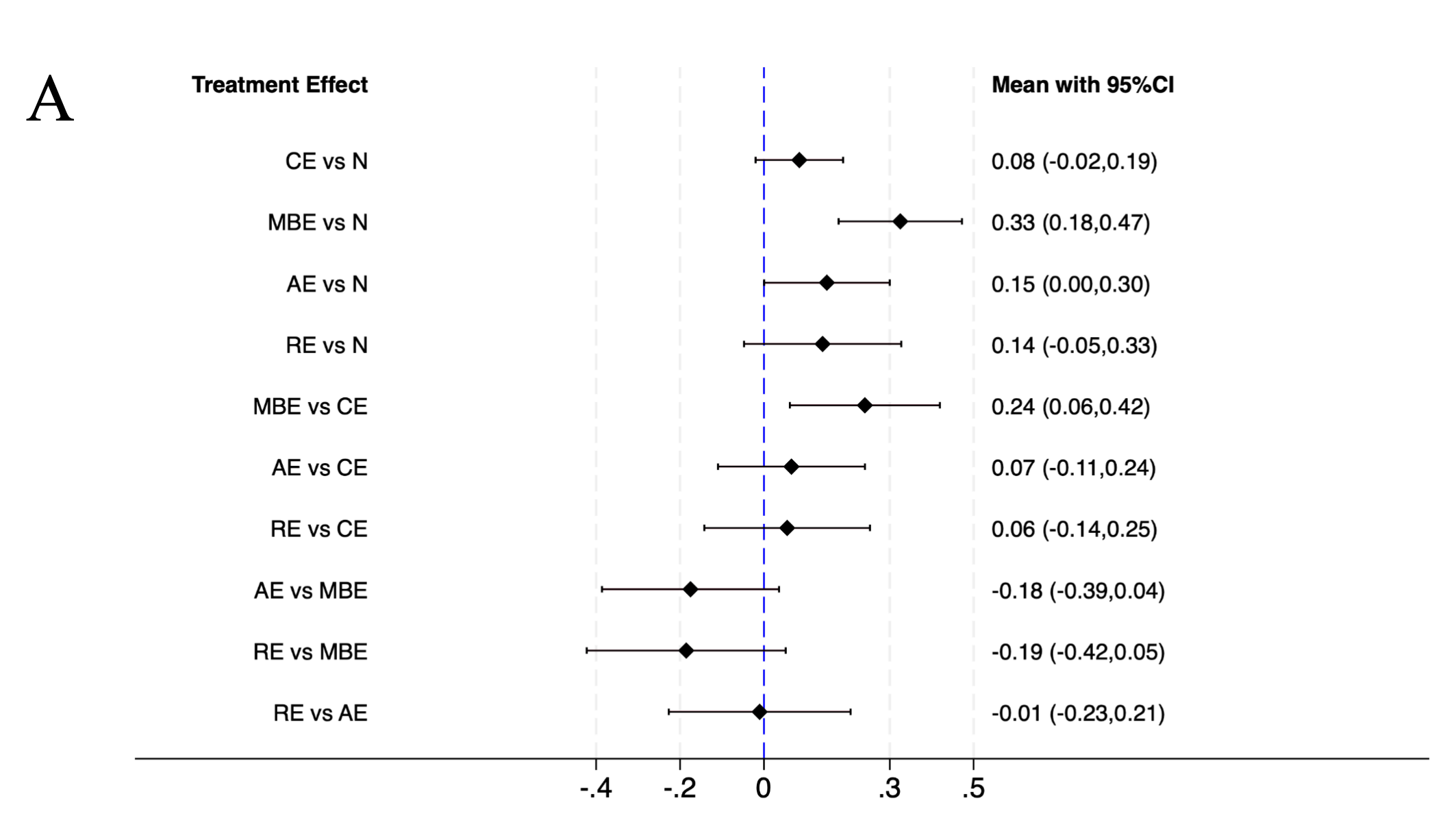


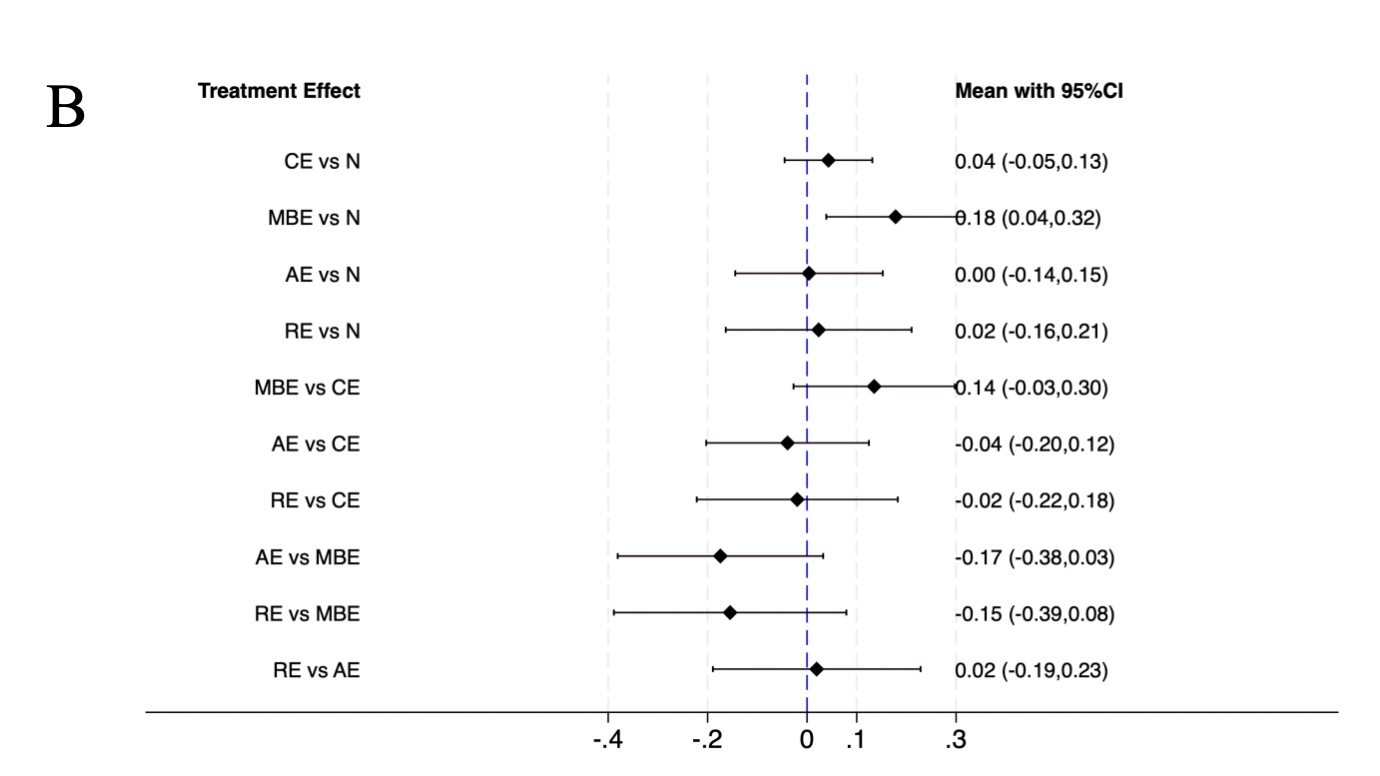


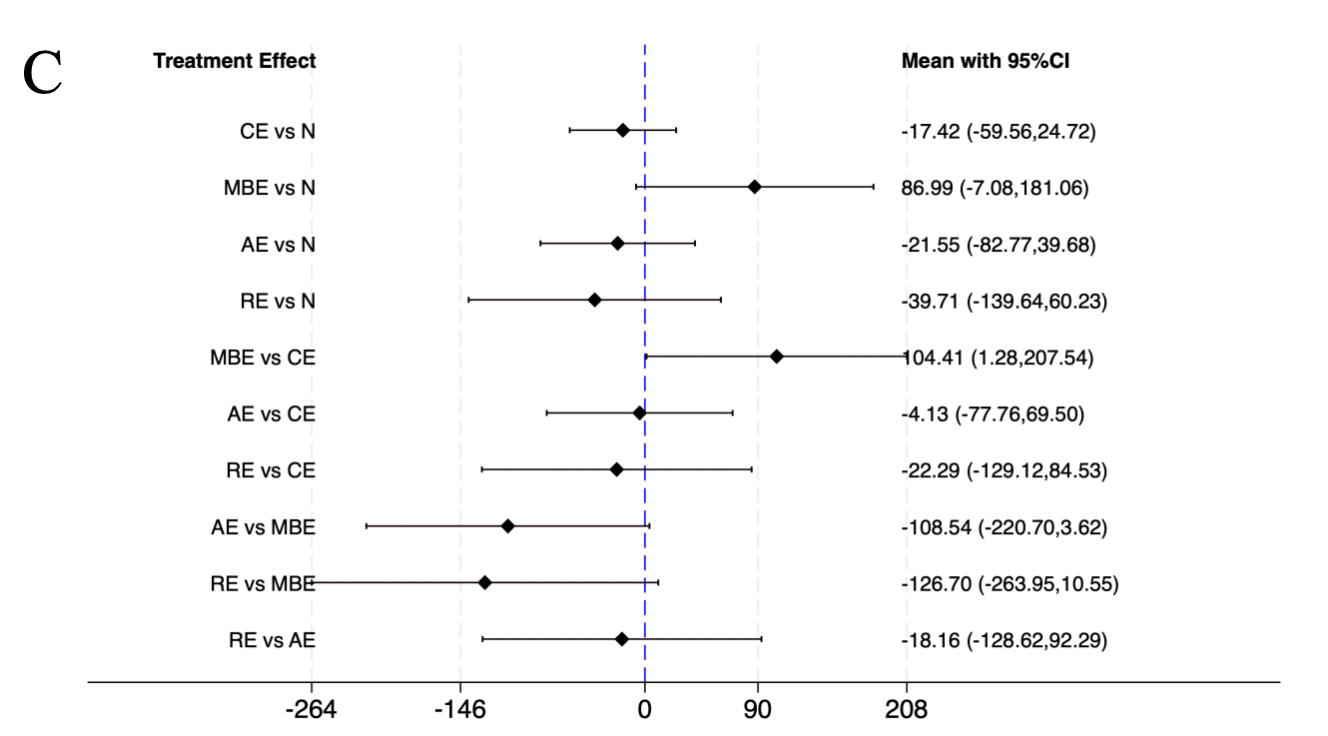


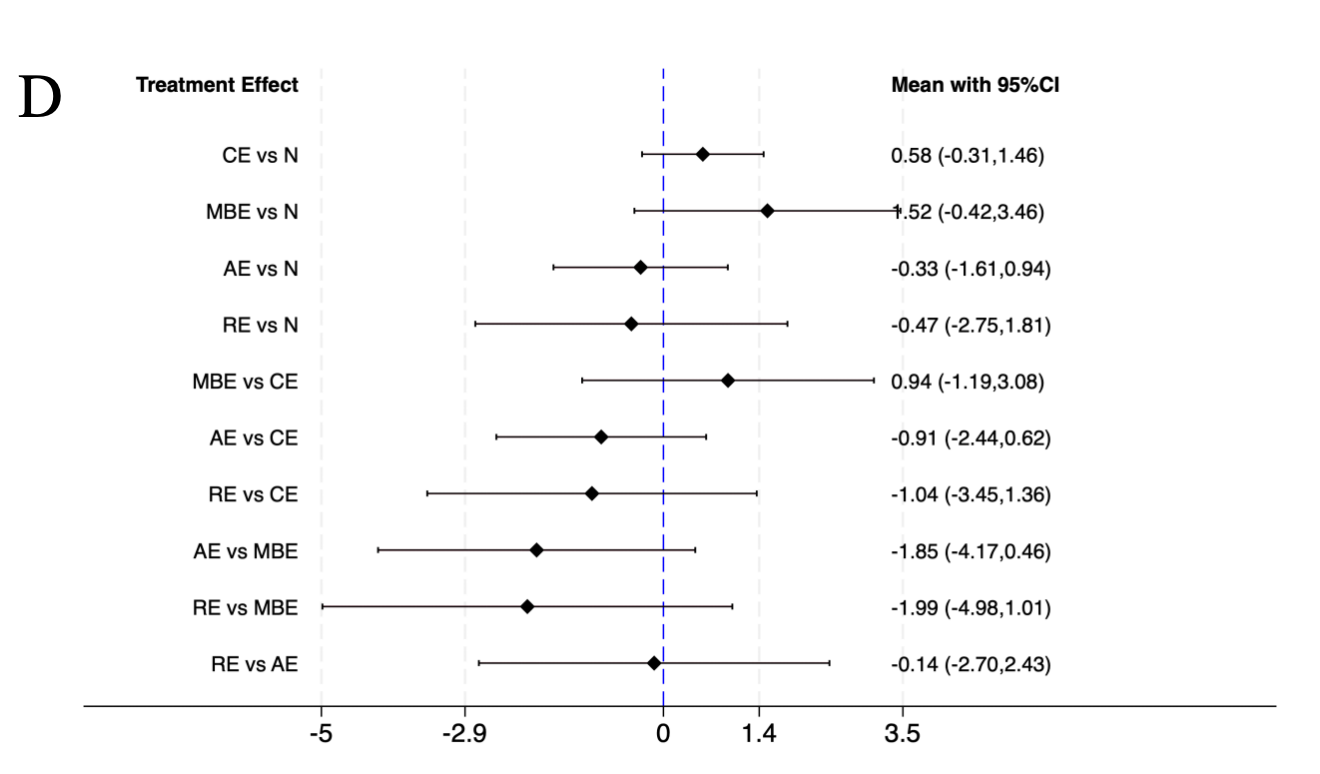


Forest plots.A: 1-minute Apgar score,B: 5-minute Apgar score，C: Birth weight，D：Gestational age.
